# Complete biosynthesis of a sulfated chondroitin in *Escherichia coli*

**DOI:** 10.1038/s41467-021-21692-5

**Published:** 2021-03-02

**Authors:** Abinaya Badri, Asher Williams, Adeola Awofiranye, Payel Datta, Ke Xia, Wenqin He, Keith Fraser, Jonathan S. Dordick, Robert J. Linhardt, Mattheos A. G. Koffas

**Affiliations:** 1 Department of Chemical and Biological Engineering, Rensselaer Polytechnic Institute, Troy, NY USA; 2 Department of Biological Sciences, Rensselaer Polytechnic Institute, Troy, NY USA; 3 Department of Chemistry and Chemical Biology, Rensselaer Polytechnic Institute, Troy, NY USA

**Keywords:** Metabolic engineering, Metabolic engineering, Applied microbiology

## Abstract

Sulfated glycosaminoglycans (GAGs) are a class of important biologics that are currently manufactured by extraction from animal tissues. Although such methods are unsustainable and prone to contamination, animal-free production methods have not emerged as competitive alternatives due to complexities in scale-up, requirement for multiple stages and cost of co-factors and purification. Here, we demonstrate the development of single microbial cell factories capable of complete, one-step biosynthesis of chondroitin sulfate (CS), a type of GAG. We engineer *E. coli* to produce all three required components for CS production–chondroitin, sulfate donor and sulfotransferase. In this way, we achieve intracellular CS production of ~27 μg/g dry-cell-weight with about 96% of the disaccharides sulfated. We further explore four different factors that can affect the sulfation levels of this microbial product. Overall, this is a demonstration of simple, one-step microbial production of a sulfated GAG and marks an important step in the animal-free production of these molecules.

## Abstract (web summary)

Chondroitin sulfate (CS) is a type of sulfated glycosaminoglycan that is manufactured by extraction from animal tissues for the treatment of osteoarthritis and in drug delivery applications. Here, the authors report the development of single microbial cell factories capable of compete, one-step biosynthesis of animal-free CS production in *E. coli*.

## Introduction

Western medicine relies heavily on sulfated glycosaminoglycans (GAGs)^1^. The two most commonly used GAGs are heparin and chondroitin sulfate (CS). Since the 1940s, heparin has predominated as the primary anticoagulant used in medicine^2^. CS, the most abundant GAG in the human body, is an important component of joint tissues and is frequently used in the treatment of osteoarthritis^3,4^. Since they are exclusively present in animals, all GAGs are currently commercially manufactured by extraction from animal tissues, primarily from bovine trachea and porcine intestinal mucosa. Prime producers of pig and cattle, such as China, dominate the manufacturing and marketing of GAGs. These sulfated polysaccharides always occur as mixtures in tissues with individual components varying slightly in stereochemistry, length, and sulfation pattern^5^. Such small analytical differences are both a blessing and a curse; they result in remarkably distinct biological function and in vivo behavior; however, they also make their adulteration very hard to detect. Contamination incidents like the heparin adulteration crisis of 2008^6–8^ and FDA’s warning about questionable crude GAG sources in 2017^9^ have evoked a major conversation about the deficiencies of current production methods, regulatory practices, and analytical detection methods of adulterants/contaminants in GAGs.

GAGs also have complicated structures necessitating sophisticated analytical instrumentation for verifying their purity^10^. GAG activity and specificity are dependent upon their functional group pattern. Specific interactions of GAGs with important biomolecules bring about their physiological roles like anticancer^11^ and anti-diabetic properties^12^. The potentials of such properties have created additional demands for the sustainable availability of pure, chemically defined GAGs. Difficulties in downstream purification, complex and expensive quality control steps, risk of cross-viral contaminations, non-sustainability, and heterogeneity in GAGs from animal tissues and cultural trends against animal-sourced products are key forces driving innovation in GAG manufacturing towards sustainable, microbial-based processes.

Chondroitin sulfate (CS), the first GAG to have been extracted and identified^13^, is extensively prescribed in human and veterinary joint health^4^. CS is composed of [→4)-β-D-GlcA-(1 → 3)-β-D-GalNAc-(1 → ] repeating disaccharide units with various combinations of sulfation and epimerization generating different CS types. CS is extracted primarily from cartilaginous tissues, with bovine trachea and shark cartilage serving as major raw materials for CS manufacturing^14,15^. Complex CS structures in proteoglycans have myriad functional group patterns that allow specific interactions with biomolecules^16^. Such interactions regulate many important cellular processes, including differentiation and development,^17–19^ and determine the role of CS in health and disease. For example, specific patterns of fucosylated CS from sea cucumbers were shown to possess anti-obesity, anti-diabetic and immunomodulatory activities^20^. Systematic studies identifying such favorable, physiological activities of specific CS types are severely limited by the availability of pure CS.

A major solution to the limitations associated with the current state-of-art CS production is production using animal-free methods^21–23^. Chemical, chemoenzymatic, microbial, and mammalian cell-culture based CS production systems are being developed worldwide^21^. Chemical synthesis methods are tedious, involving multiple steps, and are difficult to scale up. Similarly, synthesis from mammalian cell cultures is not ideal due to handling complexities, high media cost, low cell densities, and interference from other GAG pathways.

**Fig. 1:**
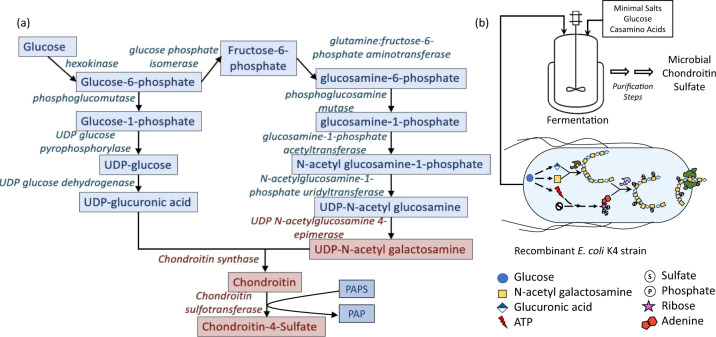
In vivo production of different CS types. **a** Biosynthetic pathway for chondroitin production from glucose. The parts marked in red are absent in common lab strains of *E. coli*. **b** Schematic of microbial CS production from minimal nutrient media using recombinant *E. coli*.

Complete biosynthesis of CS in *E. coli* requires three factors; unsulfated chondroitin precursor, the sulfate donor 3′-phosphoadenosine-5′-phosphosulfate (PAPS), and chondroitin sulfotransferase. Chondroitin and PAPS are the substrates for CS and each is produced from distinct metabolic pathways. Overall, a total of 13 metabolic reactions are required for chondroitin and PAPS synthesis. Of the 11 metabolic steps to synthesize chondroitin, nine are already present in commonly used *E. coli* strains (Fig. 1a). Enzymes for the two remaining steps (UDP-N-acetylglucosamine-/UDP-glucosamine-4-epimerase and chondroitin synthase) have been identified in *E. coli* K4 (Serovar O5:K4:H4)^24^. Incidentally, other bacteria like *Pseudomonas aeruginosa* serotype O6^25^, *Yersinia enterocolitica* serotype O8^26^, *Pasteurella multocida* Type F^27^, and *E. coli* O86:B7^28^ possessing either or both of these chondroitin-specific enzymes have also been characterized.

Complete microbial synthesis of CS holds great promise as it represents a one-step, sustainable process for the production of structurally homogeneous CS. However, this has not been practically accomplished until now. Since the enzymatic route for CS production is well known, multi-step chemoenzymatic methods have served as a placeholder for biotechnological production. *Escherichia coli* is a model organism heavily used in the production of recombinant products and non-native metabolites due to its genetic tractability and ease of scale-up. In this study, we describe the development of a metabolically engineered *E. coli* strain for the complete biosynthesis of animal-free CS (Fig. 1b). We accomplish this by identifying the factors that control CS sulfation levels and engineering efficient chondroitin sulfotransferases. This study represents a one-step, in vivo, microbial synthesis of animal-free sulfated glycosaminoglycans.

## Results

Chondroitin sulfation is catalyzed by chondroitin sulfotransferases. These enzymes transferases of animal origin that undergo posttranslational glycosylation and are present in Golgi apparatus with no identified microbial counterparts. This requires that they be expressed as heterologous proteins. While different chondroitin sulfotransferases give rise to different forms of CS, in the current study, we focus on one homolog of this class, chondroitin-4-*O*-sulfotransferase (S_w_) that results in the production of the 4-*O*-sulfated CS-A.

*E. coli* K4 was selected to assemble the required elements for CS biosynthesis. This strain has been extensively studied for chondroitin production^24,29–31^. However, K4 produces a fructosylated chondroitin as part of its capsular polysaccharide. The fructosyltransferase, responsible for fructosylation of chondroitin’s GlcA residues at the 3-position, is unfavorable for CS production. For this reason, we first deleted the fructosyltransferase-encoding gene, thereby resulting in the generation of non-fructosylated chondroitin in *E. coli* K4^32,33^. We also integrated the T7 RNA polymerase into the genomic *lacZ* site in *E. coli* K4 Δ*kfoE*, generating K4 Δ*kfoE* (DE3), to facilitate the expression of recombinant genes under the stronger T7 promoter system (Supplementary Figure 1).

### PAPS accumulation facilitates intracellular CS synthesis

**Fig. 2:**
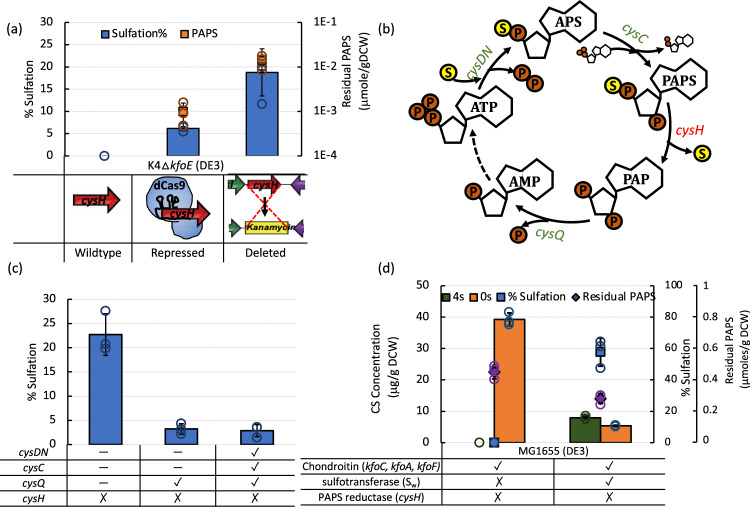
Effect of PAPS accumulation on CS sulfation. **a** CS sulfation and residual PAPS in K4Δ*kfoE*(DE3)pETM6-S_w_ strains with wildtype, repressed and deleted *cysH*. **b** Biosynthetic route for PAPS synthesis. **c** Effect of overexpression of PAPS biosynthetic enzymes on CS sulfation in K4Δ*kfoE*(DE3)pETM6-S_w_. **d** CS titer, residual PAPS and sulfation in MG1655Δ*cysH*(DE3) expressing chondroitin synthesis and sulfotransferase enzymes. (Xs = CS disaccharide sulfated in the X position of *N*-acetylgalactosamine). Error bars represent standard deviation from three biological replicates. Source data underlying Figs. 2a, c, and d are provided as a Source Data file.

In our first attempt to enable CS synthesis, we simply added the missing sulfotransferase component to K4 Δ*kfoE* (DE3) by expressing S_w_ via the pETM6-S_w_ plasmid. However, no CS synthesis was observed (Fig. 2a). Sulfation of chondroitin to CS requires PAPS, which is the universal sulfate donor responsible for most biological sulfation processes. PAPS biosynthesis is a subset of the ubiquitous cysteine/methionine biosynthetic pathways, and therefore, is present in almost all cell types, including *E. coli*. Due to the presence of high levels of chondroitin and previously demonstrated successful S_w_ expression, we hypothesized that the lack of CS production was due to limitations in intracellular PAPS^50^. PAPS biosynthesis involves a two-step activation of inorganic sulfate onto ATP catalyzed by ATP sulfurylase (*cysDN*) and APS kinase (*cysC*) (Fig. 2b). However, the native pathway also consists of PAPS reductase (*cysH*) that competes with sulfotransferases and reduces PAPS to inorganic sulfite (Fig. 2b).

To address the elimination of such a competitive enzyme expression, we adopted a *cysH* repression or deletion strategy in the K4Δ*kfoE*(DE3)pETM6-S_w_ strain and evaluated PAPS accumulation and CS sulfation to explore whether PAPS was indeed the limiting component in this strain (Fig. 2a). PAPS accumulation achieved in this strain through the manipulation of *cysH* enabled intracellular sulfation of chondroitin, demonstrating that it is indeed possible to completely biosynthesize CS in *E. coli*. This result shows that PAPS pathway intervention is a necessary condition for GAG biosynthesis in *E. coli*.

As a result of *kfoE* and *cysH* deletions and S_w_ overexpression, CS sulfation yield reached ~19%, which is lower than that in animal-extracted CS-A. Thus, there is room for improvement Overexpression of other PAPS biosynthetic genes (*cysDNCQ*) in the K4Δ*kfoE*Δ*cysH*(DE3) significantly reduced the sulfation of chondroitin (Fig. 2c). Lack of success in this strategy also suggests that using a strain that responds to *cysH* deletion with higher PAPS accumulation might lead to higher CS sulfation. Since our previously developed *E. coli* MG1655Δ*cysH*(DE3)^34^ accumulated about 54-fold higher (~0.8 μmoles/gDCW) PAPS than K4Δ*kfoE*Δ*cysH*(DE3), we also explored CS synthesis in this strain. Chondroitin production in MG1655Δ*cysH*(DE3) was enabled by expression of the K4 genes, *kfoC*, *kfoA,* and *kfoF* through the plasmid pETM6-PCAF, previously constructed in our lab^31^. Furthermore, the assembly of all three components in MG1655Δ*cysH*(DE3) by co-expression of S_w_ (pETM6-PCAFS_w_) led to a high intracellular CS sulfation level of 58% (Fig. 2d). The metabolic cost of S_w_ expression was also observed in the form of a decrease in total CS.

### Improving sulfotransferase activity enhances CS sulfation

**Fig. 3:**
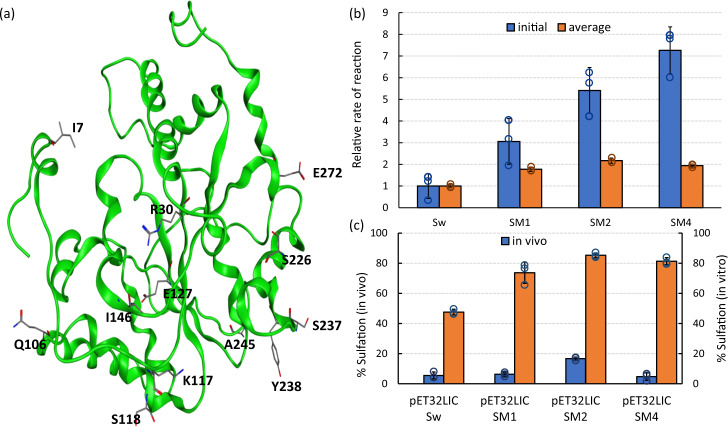
Effect of sulfotransferase variants on CS sulfation. **a** Locations of predicted mutation displayed on S_w_ structure. **b** Relative initial and average rate of reaction (in vitro) of mutant sulfotransferases compared to S_w_. **c** In vitro and in vivo (in K4Δ*kfoE*Δ*cysH*(DE3)) CS sulfation levels of S_w_ and mutants. Error bars represent standard deviation from three biological replicates. Source data underlying Fig. 3b and c are provided as a Source Data file.

Chondroitin sulfotransferases are Golgi-based, integral transmembrane glycoproteins. These enzymes must be truncated, correctly folded and expressed in a soluble form in order to be effective catalysts for *E. coli*-based CS synthesis^35^. Based on the hypothesis that increased solubility and stability of S_w_ would improve CS sulfation levels, we relied on a computational approach to identify mutations enhancing these properties. A web-based server called Protein Repair One Stop Shop (PROSS) utilizes sequence and structure information of proteins to identify residue mutations that would result in enhanced expession and solubility^36^. While the sequence of human S_w_ was known based on our previous work on its expression in *E. coli* and *P. pastoris*, a structure is not yet available in the protein databank. Hence, we constructed a homology model of S_w_ structure to use in PROSS that is based on the previously elucidated structure of the sulfotransferase domain of an olefin synthase from *Synechococcus* PCC 7002 (RMSD of 0.74). The sequence alignment of S_w_ with the template is shown in Supplementary Figure 2a. From our analysis using the PROSS server, we were able to identify three variants of S_w_, each containing multiple mutations across the sequence (Sequences in Supplementary Table 4). The mutants are named S_M1_ (H127E S238Y), S_M2_ (K117R H127E S238Y A245G), and S_M4_ (I7A R30Q Q106E K117R S118N H127E I146T S226A S237D S238Y A245G E272Q). All of the predicted mutations were at solvent accessible residues, distant from the active site of the enzyme (Fig. 3a). Mutations I7A, R30Q, Q106E, S118N, H127E, I146T, S226A, S237D, S238Y, E272Q resulted in a modified charge distribution at these solvent accessible positions (Sequence Alignments in Supplementary Figure 2a).

The three mutant enzymes (designated as S_M1_, S_M2_, S_M4_) were expressed in *E. coli* BL21(DE3) using a pET32LIC vector and were then purified. While all three mutants demonstrated enhanced in vitro sulfotransferase activity compared to S_w_ (Fig. 3b and Supplementary Fig. 7), S_M2_ demonstrated the highest conversion. Transformation of the mutant plasmids into K4Δ*kfoE*Δ*cysH*(DE3) (PAPS-accumulator and chondroitin-producer) helped verify the effect of these mutants on in vivo constructs. Expression of S_M1_ and S_M4_ did not result in significantly different intracellular sulfation levels compared to S_w_. In contrast, S_M2_ produced CS with a three-fold increased sulfation (Fig. 3c). The disparity between the in vitro and the corresponding in vivo activities (Sw(in vitro) vs. Sw(in vivo); SM1(in vitro) vs. SM1(in vivo); etc.) could be attributed to differences in the levels of expressed chondroitin-4-*O*-sulfotransferase and/or the chondroitin and PAPS substrates in the respective experiments. Another noteworthy aspect is the difference in the sulfation level of S_w_ while expressed using pET32LIC (Fig. 3c) and pETM6 (Fig. 2a). The intracellular level of sulfation obtained by pET32LIC-S_w_, was 4- to 6-fold lower than that obtained by pETM6-S_w_ depending on expression temperature (Supplementary Figure 3). While both these plasmids have similar backbones, the same origin of replication, and antibiotic resistance, the pET32LIC version expresses the enzyme with N-terminal Trx and His tags. In contrast, pETM6 expresses the enzyme sequence without the tags and is also a plasmid backbone generally used in metabolic engineering studies.

### Sulfation level is dependent on sulfotransferase induction

**Fig. 4:**
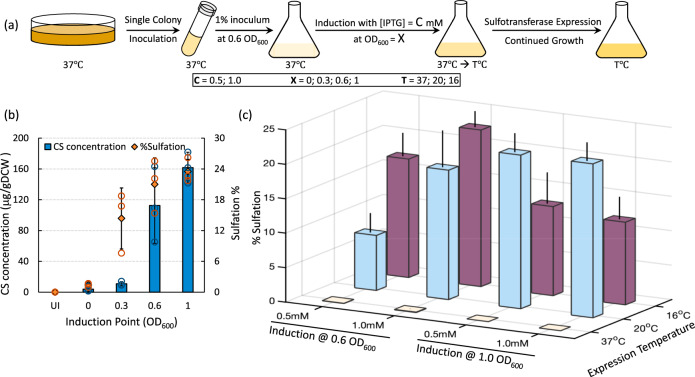
Effect of fermentation conditions on CS sulfation. **a** Schematic showing growth and cultivation of CS-producing *E. coli* strains. **b** Effect of Induction OD_600_ on CS titer and sulfation in K4Δ*kfoE*Δ*cysH*(DE3)pETM6-S_w_. **c** CS sulfation obtained from different inducer concentrations and expression temperatures for 0.6 and 1.0 OD_600_ induction points. Error bars represent standard deviation from three biological replicates. Source data underlying Figs. 4b and c are provided as a Source Data file.

Growth and induction conditions have been shown to have an effect on biosynthesis of many microbial products^37–39^. For purposes of GAG production, in vivo sulfation level is a metric of equal, or greater, importance than titer. To explore the effect of induction on in vivo sulfation, K4Δ*kfoE*Δ*cysH*(DE3) pETM6-S_w_ was grown and induced for S_w_ expression with IPTG at different growth stages (Fig. 4a). Induction at 0.6 OD_600_ and 1.0 OD_600_ was deemed optimal since they both resulted in significantly higher amounts of CS and higher levels of sulfation (Fig. 4b). Hence, both these induction points were further tested for optimal inducer concentration (0.5 mM (low) and 1.0 mM (high) [IPTG]) and optimal expression temperature (37 °C, 20 °C, and 16 °C) (Fig. 4c). Three conditions resulting in relatively high sulfation of chondroitin were identified. These were cultures induced at 1.0 OD_600_ with either low or high IPTG concentration and expressed at 20 °C for 12 h and cultures induced at 0.6 OD_600_ with high IPTG concentration and expressed at 16 °C for 24 h. In contrast to induction at 1.0 OD_600_, induction at 0.6 OD_600_ also made the culture more sensitive to inducer concentration. Overall, employing optimal induction conditions improved the CS sulfation in *E. coli* K4Δ*kfoE*Δ*cysH*(DE3) pETM6-S_w_ from ~19% to ~23%.

### Impact of cellular export mechanism on CS sulfation

**Fig. 5:**
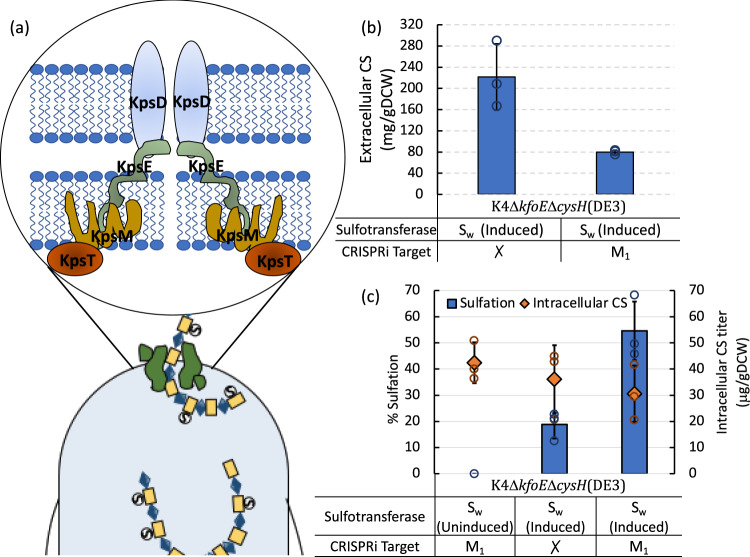
Effect of export on CS sulfation. **a** Representation of currently accepted CPS transport complex in *E. coli* producing group 2 capsules. **b** Repression of GAG transport using CRISPRi in K4Δ*kfoE*Δ*cysH*(DE3)pETM6-S_w_ showing 2.5-fold decreased GAG export. **c** CS sulfation in transport repressed K4Δ*kfoE*Δ*cysH*(DE3) expressing S_w_ with uninduced and unrepressed controls. Error bars represent standard deviation from three biological replicates. Source data underlying Figs. 5b and c are provided as a Source Data file.

*E. coli* K4 naturally exports fructosylated chondroitin through a transporter protein complex. Previous studies have shown that the majority of unsulfated chondroitin produced is exported to the medium^40^. In the current study, we observed that CS, unlike unsulfated chondroitin, was not present in the extracellular medium. Instead, all reported CS production was intracellular. While our experiments point to strategies to improve intracellular sulfation, the bulk of the polysaccharide was both secreted and completely unsulfated (Supplementary Table 1). This led us to examine the possibility that the export of the unsulfated chondroitin is in competition with its sulfation and CS production. We employed CRISPRi-based repression of the transporter genes to verify the effect of decreasing transport activity on intracellular sulfation. The currently accepted mechanism of GAG transport in *E. coli* K4 involves an ABC membrane transporter complex formed by four proteins, KpsT, KpsM, KpsD, and KpsE^41^ (Fig. 5a). KpsT is an ATPase that complexes with an inner membrane permease, KpsM. KpsD and KpsE each form dimeric periplasm and membrane-spanning complexes that facilitate the export of the polysaccharide^41^.

Five spacers targeting *kpsT* and *kpsM*, designated as dT1, dT2, dT3, dM1 and dM2, were chosen to repress GAG transport (Supplementary Fig. 4a). They were cloned into the CRISPRi plasmid pdCas9, transformed into *E. coli* K4Δ*kfoE*Δ*cysH*(DE3) pETM6-S_w_. The strain obtained by transformation with pdCas9-dM_1_ was chosen for further investigating the effects of export on sulfation since it demonstrated a 60% decrease in export (Fig. 5b) and Supplementary Fig. 4b). The sulfation of chondroitin in this strain was improved by about 3-fold to ~55%, while no significant differences were observed in the total GAG titer (Fig. 5c). This suggests that preventing GAG export results in higher levels of sulfation.

### High sulfation can be achieved by fermentation optimization

Upstream fermentation modifications of the constructed CS producer - MG1655Δ*cysH*(DE3) pETM6-PCAFS_w_ resulted in improvements in CS production. Including a second subculture and fermenting in a bioreactor with controlled pH significantly improved sulfation levels to 96.12 ± 2.55% 4-sulfated CS-A. We also saw minor improvements in titer to about 27 μg/g DCW. Molecular weight (M.W.) analysis (Supplementary Fig. 5) showed that the CS from this MG1655 CS producer is highly polydisperse, with a lower average M.W. Low M.W. CS (1000-3000 Dalton) has potential applications as dietary supplements.

## Discussion

Glycosaminoglycans are an important class of pharmaceuticals that are plagued by contamination crises, dependence on extraction from animal tissues and variable quality control^6–9^. Existing alternatives for animal-sourced GAG production are impeded by the requirement of complex cofactors, poorly active enzymes, poor expression and solubility in bacterial hosts, and difficulties in scale-up^21^. New biological roles of GAGs are still being uncovered and represent a potential paradigm change in their pharmacological applications^11,12^. Moreover, GAGs represent important co-receptors for pathogens in infectious diseases^42^. For example, the spike protein of SARS-CoV-2 virus tightly binds to the host cell GAG, heparan sulfate, leading to virus interaction with its protein-based ACE2 receptor and resulting in COVID-19^43^.

Here, we demonstrate the in vivo production of a sulfated GAG in metabolically engineered *E. coli* strains and investigate strategies for improving product levels. Engineered PAPS accumulation was shown to be necessary for in vivo sulfation of chondroitin but improving PAPS regeneration decreased the level of CS production (Figs. 2a and 2c). Optimizing pathway overexpression might overcome the metabolic burden of PAPS regeneration. There might also be unknown transcriptional and metabolic regulation in the PAPS pathway, reducing any further PAPS accumulation. We also showed that selecting a base strain (MG1655(DE3)) that accumulated higher amounts of PAPS on the deletion of *cysH* currently represents the best approach for achieving higher sulfation (Fig. 2d). Engineering PAPS accumulation in *E. coli* is still relatively unexplored and could serve beneficially to a wide range of sulfated biologics with only a couple of studies in recent years^34,44^.

A second aspect of the current study examines the role of engineering carbohydrate sulfotransferases on GAG production. These Golgi enzymes of animal origin might be further improved through synthetic biology and protein engineering for enhanced expression and activity in prokaryotic cells. While some studies describe such expression attempts with carbohydrate sulfotransferases^35,51,56^, they have only further employed them in vitro for metabolite production. In the case of chondroitin sulfotransferases, rational protein design is currently limited by the lack of structural information. This study relied on a homology model based on the known structure of a different enzyme, a sulfotransferase component of olefin synthase^53^. This enzyme has a low sequence similarity to chondroitin sulfotransferase. Despite this limitation, the PROSS server predicted improved mutants (Fig. 3). It is noteworthy that the PROSS-predicted mutations, aimed at increasing stability and solubility, provided a three-fold enhancement of in vivo CS sulfation. This highlights the potential of more highly targeted enzyme engineering to further improve in vivo sulfation.

Our study also suggests that Golgi sulfotransferases of animal origin are a challenging group of proteins to actively express and conditions such as lower temperature are needed to achieve higher intracellular sulfation of chondroitin. Continuing growth at 37 °C post-induction resulted in no CS sulfation irrespective of induction OD_600_ or inducer concentration indicating poor expression of active S_w_ at higher temperatures. Likewise, incubation at lower temperatures from the start of the fermentation resulted in slow growth and in minimal sulfation. Hence, dropping the temperature to 20 °C or 16 °C upon induction was critical for in vivo sulfation. Expression at 16 °C resulted in improved CS sulfation in cultures induced at 0.6 OD_600_, while expression at 20 °C resulted in improved sulfation for cultures induced at 1.0 OD_600_ (Fig. 4). In contrast to sulfotransferase expression, the production and export of chondroitin is relatively straightforward and is optimal at 37 °C. In addition to engineering sulfotransferases for better in vivo activity, future studies should also aim at engineering precursor production and CS export to work optimally through a range of temperatures.

Another important finding of the current study is the effect of repression of CS export on sulfation levels (Fig. 5). The GAG transporter genes *kpsT* and *kpsM* are located in region III of the capsular polysaccharide gene cluster of *E. coli* K4 under the control of a thermoregulated promoter^41^. Hence it is possible that the temperature drop used for sulfotransferase expression represses the export of CS. This temperature effect together with CRISPRi-based *kpsM* repression, could significantly impede CS export, thereby allowing more time for the sulfation of chondroitin. This is consistent with the three-fold increase in sulfation of chondroitin observed in this strain. However, this metabolic design rules out CS secretion, making the approach less attractive for scale-up and downstream processing. Future studies should take into account strain designs that can slow down export while not eliminating it completely.

We also observed that the CS sulfation levels dropped when we tried combining the successful approaches observed above. K4Δ*kfoE*Δ*cysH*(DE3) pETM6-S_M2_ pdCas9-dM1 and MG1655ΔcysH(DE3) pETM6-PCAFS_M2_ showed only about 4% and 5% sulfation of chondroitin. Two *E. coli* strains developed in this study successfully synthesized high levels of 4-*O*-sulfated chondroitin, CS-A. These were - K4ΔkfoEΔcysH(DE3) pETM6-Sw pdCas9-dM1 and MG1655ΔcysH(DE3) pETM6-PCAFSw. The CS obtained from these microbial strains is comparable to industry-standard, animal-sourced CS-A from bovine trachea. The key take-away is that the level of 4-*O*-sulfation in the animal (~70%) and microbial CS (~55%) was similar. More importantly, unlike animal-sourced CS-A, microbial CS did not contain the 6-*O*-sulfated isomer CS-C as an impurity. Details on the disaccharide compositions, along with comparisons to other commercially available CS-A are given in Supplementary Fig. 6.

Overall, this report describes prokaryotic production of a sulfated GAG by fermentation on glucose, minimal salts, sulfate, and casamino acids. This one-step, sustainable process offers a promising alternative to animal extraction in GAG production. This approach also offers the potential to prepare unnatural GAG derivatives and stable isotope-labeled GAGs^45^. In improving the precursors required for CS synthesis, we also demonstrate an increased sulfation level of the intracellular product to ~96%, comparable to commercially available CS-A. Moreover, unlike the animal-derived CS, the microbial product does not contain any impurities such as CS-C. In addition to demonstrating that sulfated GAGs can be produced entirely by metabolically engineered prokaryotes, this study also explores factors that might be improved to achieve even higher sulfation and production levels. The impact of this work can be extended, as our results suggest that this strategy can be extrapolated to develop microbial cell factories for producing other types of GAGs as well.

## Methods

### Reagents, bacterial strains and plasmids

LB Broth (Lennox), salts, and reagents required for super optimal broth with catabolite repression (SOC) were procured from MilliporeSigma (St. Louis, MO). BD Difco^TM^ M9 minimal media salts and BD Bacto^TM^ casamino acids were procured from BD Biosciences (Franklin Lakes, NJ). Standard lithium salt of 3′-phosphoadenosine-5′-phosphosulfate (PAPS) and reagents required for disaccharide labeling were bought from MilliporeSigma (St. Louis, MO). CS disaccharide standards were purchased from Iduron (Manchester, UK). High performance liquid chromatography (HPLC)-grade solvents and salts used to prepare mobile phases were procured from Fisher Scientific (Springfield, NJ).

Bacterial strains used in this study are *E. coli* DH5α, *E. coli* BL21Star(DE3), *E. coli* K-12 MG1655(DE3), and *E. coli* K4. ePathBrick vector pETM6 was used to overexpress Chondroitin and PAPS metabolic pathway genes^46^. pETM6 and pET32LIC were used to express chondroitin-4-*O*-sulfotransferase and its mutants. Transformants were selected using ampicillin resistance that is conferred by the vector backbone, followed by colony polymerase chain reaction (PCR) and Sanger sequencing. CRISPRi repression relied on pdCas9 plasmid, a gift from Luciano Maraffini, carrying a nuclease-null Cas9 from *Streptococcus pyogenes* and a sgRNA scaffold^47^. A list of all the plasmids and primers used in this study are given in Supplementary Table 2 and 3.

### Construction of *E. coli* K4 Δ*kfoE*

*E. coli* K4 (Serovar O5:K4:H4) was engineered for the synthesis chondroitin. The fructosyltransferase encoded by *kfoE* was deleted by λ red recombineering techniques^48^ resulting in K4 Δ*kfoE*. The FRT-flanked kanamycin resistance cassette was PCR amplified from pKD4 by deletion primers (Supplementary Table 3) with 40 nucleotides homologous regions near *kfoE* on the genome. The PCR product was purified by PCR cleanup kit (Cycle Pure Kit, Omega) and transformed into the λ red recombinase expressing *E. coli* K4. Positive knockout strains were screen by colony PCR and the transformed with pCP20, which expressed the flippase recombination enzyme, to remove the antibiotics resistance marker.

T7 RNA polymerase gene with lacUV5 promoter was integrated into the *LacZ* position in the *E. coli* K4 genome using the method described by Cox and coworkers^49^. Briefly, a small fragment of “landing pad” with a tetracycline resistant marker was amplified from pTKS/CS with flanking 40 bp homologous regions of LacZ. Transformation of this purified linear DNA into K4 Δ*kfoE* expressing λ red recombinase enabled recombination and integration. Positive colonies were verified for successful integration of the landing pad. Next, T7 RNA-polymerase gene was cloned into the pTKIP vector and transformed into K4 Δ*kfoE* strains with landing-pad integration harboring pKDRED expressing yeast restriction enzyme I-SecI. Induction of I-SecI cuts at the landing pad and also cleaves out the T7-RNA-polymerase insert from pTKIP which is finally integrated into the landing pad region with the aid of λ red recombinases. This resulted in strain *E. coli* K4 Δ*kfoE* (DE3).

### Deletion of PAPS reductase from K4 and MG1655

The *cysH* gene in *E. coli* encoding for PAPS reductas, was deleted using lambda red recombinase by the method described by Datsenko and Wanner^48^. Briefly, a linear kanamycin resistance cassette with 40-bp homology arms to the two ends flanking the chromosomal *cysH* gene was amplified from pKD4 and transformed into host expressing recombinases from pKD46. On recombination, correctly deleted colonies were selected based on: kanamycin resistance; loss of ability to grown on M9 media (without casamino acids); size of chromosomal amplicon around the *cysH* gene region; and Sanger sequencing of the amplicon. Using this method, the *cysH* gene was deleted from *E. coli* strains K4Δ*kfoE*(DE3) and MG1655(DE3).

### Microbial growth conditions

Plate cultures of *E. coli* were grown by streaking glycerol stocks (frozen) onto LB agar plates with appropriate antibiotics. Starter cultures (5 mL) were grown in LB broth by shaking with antibiotics at 37 °C in 14 mL culture tubes until growth reached OD_600_ of 0.6-0.8 (about 6 h). Flask cultures of chondroitinase and sulfotransferase producing strains were grown in 1 L of M9 medium supplemented with 80 µg/mL ampicillin in PYREX Fernbach Culture Flasks (Corning Life Sciences). Flask cultures of CS-producing strains were grown in 125 mL Erlenmeyer flasks by inoculating 1% starter culture in 25 mL of M9 media supplemented with 1% glucose, 1% casamino acids and containing the appropriate antibiotics. Cellular growth was estimated using optical density of culture at 600 nm in a Biotek plate reader. Cells were grown at 37 °C until reaching an OD_600_ of 0.6 and induced with 1 mM isopropyl-1-thio-β-D-galactopyranoside (IPTG), after which growth was continued at either 16 °C for 24 h or 20 °C for 12 h or 37 °C for 10 h. All liquid cultures were incubated in a rotary air shaker (NewBrunswick Scientific Innova 44 R) at 37 °C, 225 rpm. All CS-producing flask experiments were performed in triplicate.

### Bioreactor conditions

Fed-batch fermentations were performed in 1-L Applikon bioreactors. The fermentations consisted of a batch phase, followed by a fed-batch phase. The seed media are LB-Lennox broth and composed of tryptone (10 g/L), yeast extract (5 g/L), sodium chloride (5 g/L), and required antibiotics. The pH was adjusted to 7.0 ± 0.05. Overnight cultures (starter culture) were incubated at 37 °C (220 rpm). The starter culture was inoculated in seed 1 and the cells were grown at 37 °C (220 rpm) to a cell density (OD_600_) of 0.6. The seed 2 cells were inoculated (10–15% by vol) in batch media. The batch media was composed of tryptone (8–12 g/L, typically 10 g/L), yeast extract (10-20 g/L typically, 17 g/L), glucose (10-20 g/L, typically 20 g/L), casein hydrolysate (8–15 g/L, typically 10 g/L), MgSO_4_.7H_2_O (0–2 g/L, typically, 1.2 g/L), and required antibiotics. The pH was maintained at 6.9 ± 0.1, with 5 M NaOH. Feed was initiated when (OD_600_) reached 8.0 ± 2.5 at an initial rate of 2.5 mL/h/L and the feeding rate was adjusted, based on fluctuations in pH and dissolved oxygen (DO) levels. In some experiments, the feed was initiated 5 h EFT. The cells were fed with casamino acid solution (30-70 %, typically, 50 %) supplemented with glucose (300-500 g/L. typically 350 g/L), magnesium sulfate heptahydrate, (10-20 g/L, typically 12 g/L), yeast extract (0-100 g/L, typically, 50 g/L), and tryptone (0-100 g/L, typically 10 g/L). The sterilized (autoclaved) feed solution was maintained at 37 °C (during the feeding stage). The cell growth was monitored using OD_600_ reading using a spectrophotometer and proBE 3000 Fiber Optic Total Biomass Sensor (BE3000) (BugLab, Concord, CA 94521, USA). The cells were grown at 37 °C for 10-15 EFT. When the OD_600_ reached 22 (6.9–8.6 g/L CDW), the temperature was reduced to 16 °C. The cells were induced with 2 mM IPTG at 16 °C for 16–22 h. The dissolved oxygen was maintained using agitation (400–800 rpm), and air (0.5–1 LPM). Post-induction, the cells were harvested by centrifugation at 4000–4500 × *g* for 10–20 min at 4 °C. The cell paste was weighed and stored in Ziploc freezer bags at −80  °C.

### Molecular weight analysis

The molecular weight (MW) analysis of intact chondroitin sulfate (CS) chains was performed using a 15% carbohydrate gel, using a previously described method^52^ with some modifications. Briefly, the CS was purified from the cells and freeze dried. The freeze-died sample was dissolved in HPLC-grade water and approximately 10–30 μg of chondroitin sulfate were loaded in each well. The gel was subjected to electrophoresis at 200 V for 20 min, washed at room temperature in DI water for 30 min, stained in Alcian blue solution for 15–30 min, and destained with several washes of DI water until the gel background was clear. Gel images were acquired using CanoScan LiDE 700 F at 600 dpi and processed for densitometry data using the Un-Scan-it gel 7.1 software (Silk Science Inc., Orem, UT, USA). The data were used to calculate the weight-averaged molecular weights (*M*_W_), number averaged molecular weights (*M*_N_), molecular weight ranges (*M*_R_) and polydispersities (P).

### Repression of PAPS reductase and GAG export using CRISPRi

CRISPRi was used to repress the expression of three genes—*cysH* encoding PAPS reductase, *kpsM* encoding the permease component of the capsular export complex and *kpsT* encoding the ATPase component for the capsular transport protein. pdCas9-mCherry^47^ was cloned to incorporate spacer sequences into BsaI sites (golden gate cloning^40^). Spacer sequences were selected based on the region just before the start codon of the genes containing the 5′-NGG PAM sequence for (d)Cas9. Successful clones were selected based on chloramphenicol resistance, colony color and sanger sequencing. Sequences (Supplementary Table 3) and locations (Supplementary Fig. 4) of the spacers tested are included in the supporting information.

### Computational protein redesign of sulfotransferase

The PROSS protein engineering server was used to identify mutations to improve the sulfotransferase^36^. PROSS predicts mutations that improve protein stability through modification of protein features such as core packing, surface polarity, and backbone rigidity. We used a human chondroitin-4-*O*-sulfotransferase (S_w_) sequence with a 60 amino acid truncation in the N-terminus, previously reported by our group^51^, to build a homology model structure in the Molecular Operating Environment (MOE) software suite (Chemical Computing Group ULC, Montreal, QC, Canada) using the structure of the sulfotransferase domain from *Synechococcus* PCC 7002 Olefin Synthase (PDB code: 4GOX)^53^ as a template. Sequence alignment was generated between S_w_ and 4GOX to assess the similarity between the two sequences. Homology modeling tool in MOE generated 10 models with the following parameters enabled: C-terminal and N-terminal outgap modeling, automatic disulfide bond detection, and side-chain sampling set at 300 K using an Amber 10:EHT force field. Structural alignments and the Ramachandran statistics calculated for the models were used to assess how well the predicted structure conformed to the 4GOX structure and generally well-folded proteins.

### Sulfotransferase mutant expression and purification

The three PROSS-predicted mutants of S_w,_ designated as S_M1_, S_M2,_ and S_M4_, were examined for improved activity in *E. coli*. Mutants S_M1_ and S_M2_ were derived from S_w_ in pET32LIC through multiple rounds of site-directed mutagenesis, while the S_M4_ gene was synthesized by IDT. The genes were cloned into the BamHI and XhoI sites of a pET32LIC vector with N-terminal thioredoxin (Trx) tag (to increase protein solubility) and His-6x tag (for purification). The fusion proteins were estimated to be ~53 kDa with and PI value of 6.85 (ExPASy). The constructed plasmids were sequence verified and transformed into *E. coli* BL21Star (DE3). Overnight culture (20 mL) was centrifuged at 6800 × *g* for 10 min at 25 °C and the pellet re-suspended in 1 L of M9. Sulfotransferase expression was induced at an OD_600_ of ~0.8 with 0.2 mM IPTG and the culture was incubated for 16–20 h at 22 °C.

Cells were harvested by centrifugation at 5000 × *g* for 10 min at 4 °C and the pellet was sonicated upon re-suspension in 20 mL of 50 mM Tris-HCl buffer (pH 8.0, 500 mM NaCl, 30 mM imidazole). Cell debris was removed by centrifugation at 16,000 × *g* for 1 h at 4 °C. Cell lysate was filtered and applied to a column containing Ni-NTA resin (Qiagen) and washed with buffer A (50 mM Tris-HCl 500 mM NaCl, 30 mM imidazole pH 7.5) and eluted with buffer B (50 mM Tris-HCl 500 mM NaCl, 300 mM imidazole pH 7.5). The imidazole was removed by buffer exchange and replaced with storage buffer (50 mM Tris-HCl 500 mM NaCl, 10% glycerol pH 7.5) and kept at −80 °C until needed. S_w_, S_M1_, S_M2,_ and S_M4_ were expressed and purified under identical conditions. The expression level and the purity of the target proteins were verified by SDS-PAGE using a NuPage 10% Bis–Tris Midi gel (Invitrogen).

### PAPS estimation using HPLC/UV

On harvesting, cells were pelleted at 4 °C. Metabolites, including PAPS, were extracted from the pellet with two 30 min washes of 80% methanol solution at −80 °C. Pooled extracts could be stored at −20 °C until further analysis. PAPS concentration in the extract was estimated by HPLC using a 150 × 2 mm Develosil C-30 RPAqueous column (manufactured by Nomura Chemicals, Japan and purchased from Phenomenex, Inc., USA) in an Agilent LC1260 instrument. Potassium phosphate buffer (100 mM, pH 5.8) and 75% acetonitrile (in H_2_O) were used as mobile phases A and B respectively. The samples were run on a 40 min protocol (adapted from Furuno and co-workers^54^) at an overall flow rate of 0.2 mL/min. The gradient program was set as follows: 0% B from 0 to 10 min; 0-50% B (linear ramp) from 10 to 12 min; 50% B from 12 to 17 min; 50 to 0% B (linear ramp) from 17 to 20 min and 0% B from 20 to 40 min. Standard PAPS (detected using PDA detector at 260 nm) diluted in mobile phase A elutes at 3.1 min.

### GAG extraction and disaccharide analysis using LC/MS

Extracellular GAGs produced in each flask culture were recovered in the solution phase (spent media) after centrifugation. Intracellular GAGs were recovered by re-suspending the cell pellet, autoclaving to prepare cell lysate, and centrifuging to recover the soluble phase. Both solutions containing extracellular and intracellular GAGs were precipitated with 4 volumes ethanol and stored at −20 °C for 12 h in an explosion-proof freezer. The precipitates were collected, dried, and re-dissolved in 0.2 volume sterile water to generate GAG extracts that were stored at −20 °C until further use.

Extracted GAG solutions (100 μL) were passed through a 3 kDa spin column to remove small molecules and to exchange with digestion buffer (50 mM ammonium acetate, 2 mM CaCl_2_ (pH 7.4)). GAG solutions were added to 200 μL of digestion buffer and 20 mU purified chondroitinase ABC (25 mM Tris, 500 mM NaCl, 300 mM imidazole buffer (pH 7.4)) and incubated at 37 °C for 12 h for depolymerization. The resulting disaccharides were passed through a 3-kDa spin column, then the filtrate was collected and lyophilized. The freeze-dried disaccharide samples were fluorescently labeled by dissolving in 10 μL of a 0.1 M 2-aminoacridone (AMAC) (17:3 of dimethyl sulfoxide:acetic acid (v:v)). After incubation for 10 min at room temperature, the reaction mixture was supplemented with 10 μL of 1 M NaBH_3_CN, vortex-mixed, and incubated at 45 °C for 1 h. Samples were centrifuged and the supernatant containing the labeled disaccharides was analyzed. The AMAC-labeled disaccharides were separated by HPLC on an Agilent Poroshell 120, EC-C18 column (Agilent Technologies, Inc. Wilmington, DE) using an Agilent 1200 HPLC system with detection by a TSQ Quantum triple quadrupole electron-spray ionization mass spectrometer (Thermo Finnigan, San Jose, CA)^55^. Data were processed to identify disaccharide levels using the Thermo Xcalibur software.

### In vitro sulfotransferase assays

Colorimetric activity assay followed a previously published method with some adaptations^56^. Briefly, the total assay volume was 200 μL, consisting of 100 μL 50 mM 2-(N-morpholino)ethanesulfonic acid (MES) buffer, 20 μL *p*-nitrophenyl sulfate (PNPS) (20 mM), 20 μL chondroitin (1 mg/mL), 20 μL of 1 mg/mL AST-IV, 20 μL purified C4ST (~1 mg/mL), and 20 μL PAPS (2.5 mM). The assay solution was mixed, with PAPS added immediately before absorbance measurements were started. The temperature controlled SpectraMax plate reader (Molecular Devise, Sunnyvale, CA) was pre-incubated at 37 °C, then the formation of PNP was detected at absorbance 400 nm. The reactions were allowed to continue at 37 °C overnight, then processed for disaccharide analysis.

### Reporting summary

Further information on research design is available in the Nature Research Reporting Summary linked to this article.

## Supplementary information

Supplementary Information

Peer Review

Reporting Summary

## Data Availability

Data supporting the findings of this work are available within the paper and its Supplementary Information files. A reporting summary for this article is available as a Supplementary Information file. The datasets generated and analyzed during the current study are available from the corresponding author upon request. Source data are provided with this paper.

## Source data

Source Data

## References

[1] Köwitsch A, Zhou G, Groth T (2018). Medical application of glycosaminoglycans: a review: medical application of glycosaminoglycans. J. Tissue Eng. Regen. Med..

[2] Franchini, M., Liumbruno, G. M., Bonfanti, C. & Lippi, G. Carrion’s disease after blood transfusion. *Blood Transfusion*10.2450/2015.0096-15 (2015).

[3] Bishnoi M, Jain A, Hurkat P, Jain SK (2016). Chondroitin sulphate: a focus on osteoarthritis. Glycoconj. J..

[4] Henrotin Y, Mathy M, Sanchez C, Lambert C (2010). Chondroitin sulfate in the treatment of osteoarthritis: from in vitro studies to clinical recommendations. Therapeut. Adv. Musculoskel..

[5] Rogers, C. J. & Hsieh-Wilson, L. C. in *Carbohydr. Microarrays* (ed. Chevolot, Y.) vol. 808, 321–336 (Humana Press, 2012).

[6] Liu H, Zhang Z, Linhardt RJ (2009). Lessons learned from the contamination of heparin. Nat. Prod. Rep..

[7] Kishimoto TK (2008). Contaminated heparin associated with adverse clinical events and activation of the contact system. N. Engl. J. Med..

[8] Laurencin CT, Nair L (2008). The FDA and safety—beyond the heparin crisis. Nat. Biotechnol..

[9] Brennan, Z. *Chinese Heparin Contamination Questions Return With New FDA Warning Letter* (Regul. FocusTM, 2017).

[10] Kubaski F (2017). Glycosaminoglycans detection methods: applications of mass spectrometry. Mol. Genet. Metabol..

[11] Ahn MY (2019). Anti-cancer effect of dung beetle glycosaminoglycans on melanoma. BMC Cancer.

[12] Chen Y (2019). Glycosaminoglycan from Apostichopus japonicus improves glucose metabolism in the liver of insulin resistant mice. Mar. Drugs.

[13] Levene PA, La Forge FB (1913). On chondroitin sulphuric acid. J. Biol. Chem..

[14] Volpi N (2004). Disaccharide mapping of chondroitin sulfate of different origins by high-performance capillary electrophoresis and high-performance liquid chromatography. Carbohydr. Polym..

[15] Volpi N (2009). Quality of different chondroitin sulfate preparations in relation to their therapeutic activity. J. Pharm. Pharmacol..

[16] Shipp EL, Hsieh-Wilson LC (2007). Profiling the sulfation specificities of glycosaminoglycan interactions with growth factors and chemotactic proteins using microarrays. Chem. Biol..

[17] Djerbal L, Lortat-Jacob H, Kwok J (2017). Chondroitin sulfates and their binding molecules in the central nervous system. Glycoconj. J..

[18] Izumikawa T, Sato B, Kitagawa H (2015). Chondroitin sulfate is indispensable for pluripotency and differentiation of mouse embryonic stem cells. Sci. Rep..

[19] Sirko S, von Holst A, Wizenmann A, Gotz M, Faissner A (2007). Chondroitin sulfate glycosaminoglycans control proliferation, radial glia cell differentiation and neurogenesis in neural stem/progenitor cells. Dev..

[20] Zhu Z (2018). Sulfated polysaccharide from sea cucumber and its depolymerized derivative prevent obesity in association with modification of gut microbiota in high-fat diet-fed mice. Mol. Nutr. Food Res..

[21] Badri A, Williams A, Linhardt RJ, Koffas MA (2018). The road to animal-free glycosaminoglycan production: current efforts and bottlenecks. Curr. Opin. Biotechnol..

[22] Rondanelli M (2019). Effectiveness of non-animal chondroitin sulfate supplementation in the treatment of moderate knee osteoarthritis in a group of overweight subjects: a randomized, double-blind, placebo-controlled pilot study. Nutrients.

[23] Glass CA (2018). Recombinant heparin—new opportunities. Front. Med..

[24] Zhu H-M (2018). KfoA, the UDP-glucose-4-epimerase of Escherichia coli strain O5:K4:H4, shows preference for acetylated substrates. Appl. Microbiol. Biotechnol..

[25] Bélanger M, Burrows LL, Lam JS (1999). Functional analysis of genes responsible for the synthesis of the B-band O antigen of Pseudomonas aeruginosa serotype O6 lipopolysaccharide The GenBank accession number for the sequence reported in this paper is AF035937. Microbiology.

[26] Bengoechea JA (2002). Functional characterization of Gne (UDP-N-Acetylglucosamine- 4-Epimerase), Wzz (chain length determinant), and Wzy (O-Antigen Polymerase) of Yersinia enterocolitica Serotype O:8. J. Bacteriol..

[27] Cunneen MM, Liu B, Wang L, Reeves PR (2013). Biosynthesis of UDP-GlcNAc, UndPP-GlcNAc and UDP-GlcNAcA Involves Three Easily Distinguished 4-Epimerase Enzymes, Gne, Gnu and GnaB. PLoS ONE.

[28] Guo H, Li L, Wang PG (2006). Biochemical characterization of UDP-GlcNAc/Glc 4-epimerase from Escherichia coli O86:B7 †. Biochemistry.

[29] Xu S (2019). Chain structure and immunomodulatory activity of a fructosylated chondroitin from an engineered Escherichia coli K4. Int. J. Biol. Macromol..

[30] Zhang Q (2018). Enhancing fructosylated chondroitin production in Escherichia coli K4 by balancing the UDP-precursors. Metabol. Eng..

[31] He W (2015). Production of chondroitin in metabolically engineered E. coli. Metabol. Eng..

[32] Liu J (2014). KfoE encodes a fructosyltransferase involved in capsular polysaccharide biosynthesis in Escherichia coli K4. Biotechnol. Lett..

[33] Trilli, A., Busiello, I., Daly, S. & Bagatin, F. Biotechnological production of chondroitin. US Patent 8609394B2 (2014).

[34] Badri A, Williams A, Xia K, Linhardt RJ, Koffas MAG (2019). Increased 3′‐phosphoadenosine‐5′‐phosphosulfate levels in engineered Escherichia coli cell lysate facilitate the in vitro synthesis of chondroitin sulfate a. biotechnol. J.

[35] Nagai N (2004). Stem domains of heparan sulfate 6-O-sulfotransferase are required for Golgi localization, oligomer formation and enzyme activity. J. Cell Sci..

[36] Goldenzweig A (2016). Automated structure- and sequence-based design of proteins for high bacterial expression and stability. Mol. Cell.

[37] Jones JA, Koffas MAG (2016). Optimizing metabolic pathways for the improved production of natural products. Meth. Enzymol..

[38] Ahmadi MK, Pfeifer BA (2016). Improved heterologous production of the nonribosomal peptide-polyketide siderophore yersiniabactin through metabolic engineering and induction optimization. Biotechnol. Progr..

[39] Jones JA (2015). ePathOptimize: a combinatorial approach for transcriptional balancing of metabolic pathways. Sci. Rep..

[40] He, W. Metabolic Engineering and Applied Enzymology for the Preparation of Nutraceutical/ Pharmaceutical Chondroitin Sulfate. PhD thesis, Rensselaer Polytechnic Insitute (2017).

[41] Haas, E. K. *Analyses Of The Proteins Kpsm, Kpse And Kpsd In The Group 2 Capsular Polysaccharide Export Complex Of Escherichia Coli* (University of Manchester, 2012).

[42] Kamhi E, Joo EJ, Dordick JS, Linhardt RJ (2013). Glycosaminoglycans in infectious disease: Glycosaminoglycans in infectious disease. Biol. Rev..

[43] Kim, S. Y. et al. Glycosaminoglycan binding motif at S1/S2 proteolytic cleavage site on spike glycoprotein may facilitate novel coronavirus (SARS-CoV-2) host cell entry. *bioRxiv*10.1101/2020.04.14.041459 (2020).

[44] Chu LL (2018). Metabolic engineering of escherichia coli for enhanced production of naringenin 7-sulfate and its biological activities. Front. Microbiol..

[45] Cress BF (2019). Heavy heparin: a stable isotope‐enriched, chemoenzymatically‐synthesized, poly‐component drug. Angew. Chem. Int. Ed..

[46] Xu P, Vansiri A, Bhan N, Koffas MAG (2012). ePathBrick: a synthetic biology platform for engineering metabolic pathways in E. coli. ACS Synth. Biol..

[47] Bikard D (2013). Programmable repression and activation of bacterial gene expression using an engineered CRISPR-Cas system. Nucleic Acids Res..

[48] Datsenko KA, Wanner BL (2000). One-step inactivation of chromosomal genes in Escherichia coli K-12 using PCR products. Proc. Natl Acad. Sci. USA.

[49] Kuhlman TE, Cox EC (2010). Site-specific chromosomal integration of large synthetic constructs. Nucleic Acids Res..

[50] Engler C, Kandzia R, Marillonnet S (2008). A one pot, one step, precision cloning method with high throughput capability. PLoS ONE.

[51] He W (2017). Expression of chondroitin-4-O-sulfotransferase in Escherichia coli and Pichia pastoris. Appl. Microbiol. Biotechnol..

[52] Ly Mellisa (2011). Analysis of E. coli K5 capsular polysaccharide heparosan. Anal. Bioanal. Chem..

[53] McCarthy JG (2012). Structural basis of functional group activation by sulfotransferases in complex metabolic pathways. ACS Chem. Biol..

[54] Imamura M, Kumagai T, Sugihara N, Furuno K (2003). High-performance liquid chromatographic assay of 3′-Phosphoadenosine 5′-phosphosulfate (PAPS) and UDP-glucuronic acid (UDPGA) in cultured hepatic cell extracts. J. Health Sci..

[55] Yang B, Chang Y, Weyers AM, Sterner E, Linhardt RJ (2012). Disaccharide analysis of glycosaminoglycan mixtures by ultra-high-performance liquid chromatography–mass spectrometry. J. Chromatogr. A.

[56] Sterner E (2014). Assays for determining heparan sulfate and heparin O-sulfotransferase activity and specificity. Anal. Bioanal. Chem..

