# Supplementary material for: Complete biosynthesis of a sulfated chondroitin in Escherichia coli
Source: Nat Commun. 2021 Mar 2;12:1389. doi: 10.1038/s41467-021-21692-5 (PMC7925653; doi:10.1038/s41467-021-21692-5)
Supplement: Supplementary file 1 — Supplementary Information [file 41467_2021_21692_MOESM1_ESM.pdf]

Complete biosynthesis of a sulfated chondroitin in *Escherichia coli*

Badri *et al.*

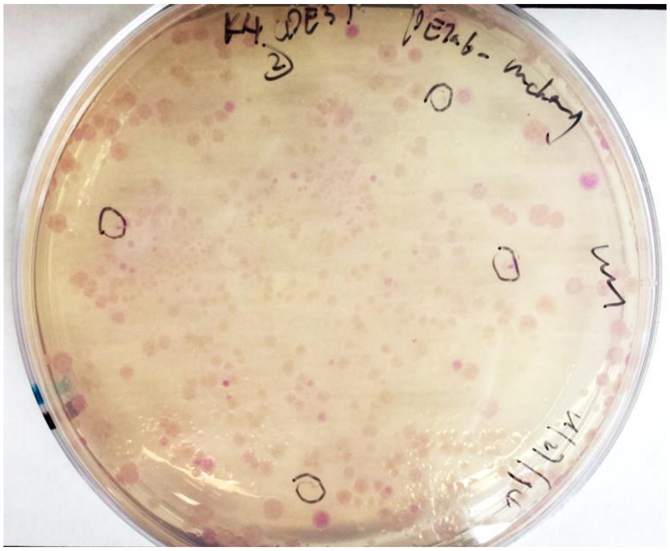

Supplementary Figure 1. *E. coli* K4 Δ*kfoE* (DE3) harboring pETM6-mCherry (under T7 promoter) on LB plate. Successful expression by the integrated T7 RNA polymerase is seen through the presence of red colored colonies.

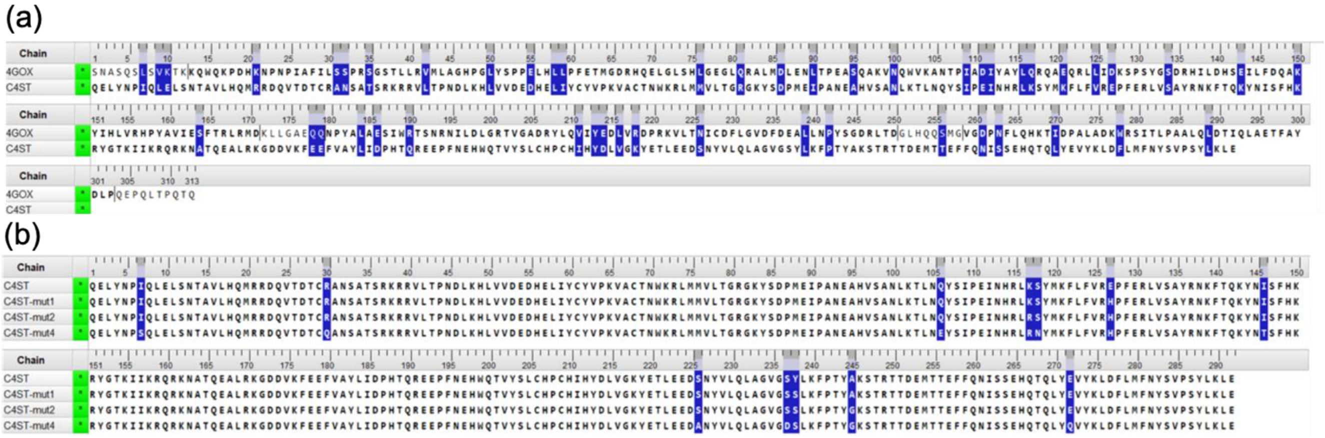

Supplementary Figure 2. Sulfotransferase sequence alignments. (a) Sequence alignment between S_w (C4ST) and sulfotransferase component of olefin synthase (4GOX). (b) Sequence alignment between S_w and the three mutants generated.

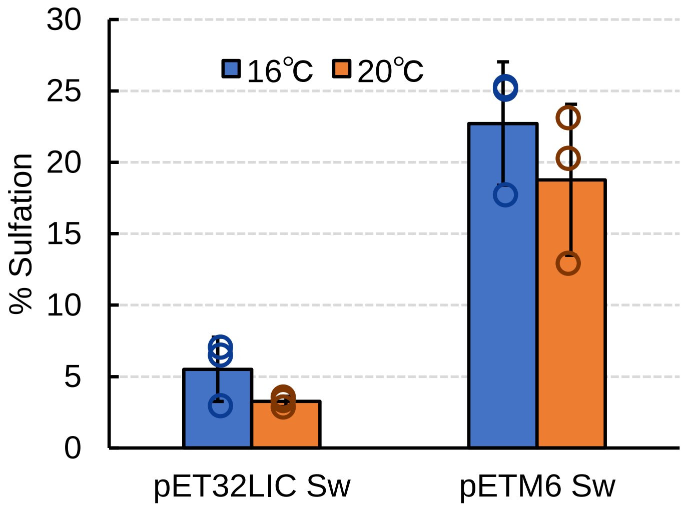

Supplementary Figure 3. Difference in CS sulfation by *K4 ΔkfoE ΔcysH* (DE3) expressing S_w using pETM6 and pET32LIC plasmids at 16°C and 20°C. Source data are provided as a Source Data file.

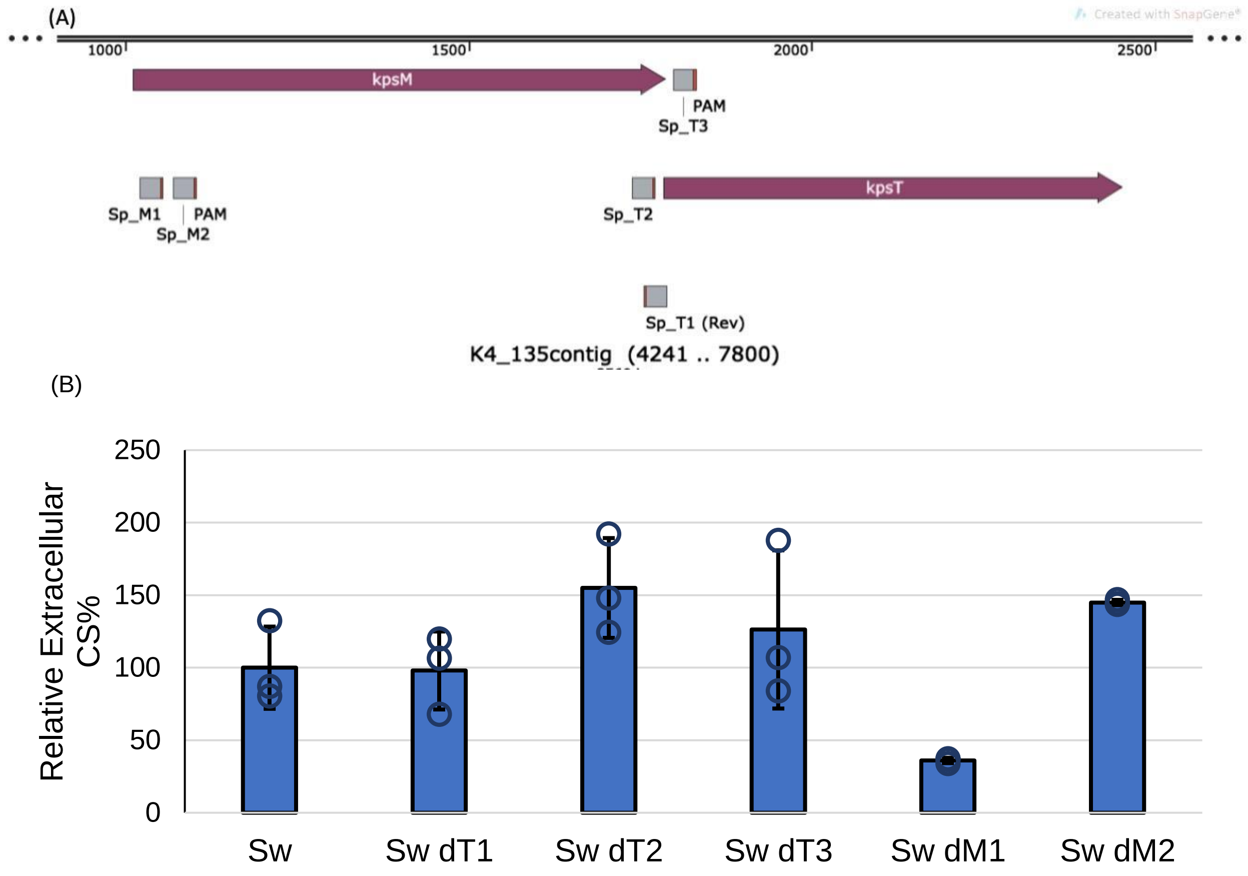

Supplementary Figure 4. CRISPRi mediated repression of GAG transport (A) Locations of *kpsM* and *kpsT* on E. coli K4's genome showing the spacers chosen for repression. (B) Relative extracellular CS of transport repression constructs to K4Δ*kfoE*Δ*cysH*(DE3)-S_w. Source data underlying Supplementary Figure 4b are provided as a Source Data file.

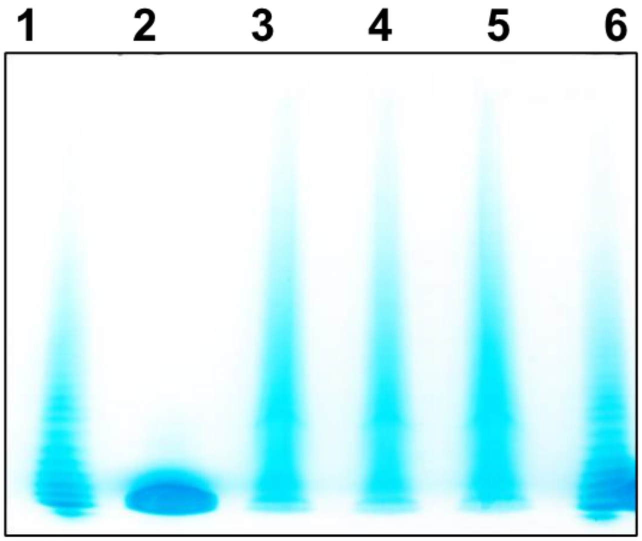

	Average of 3 lanes	S.D. of 3 lanes
Upper limit	1.12E+08	7.38E+07
Average MW	6.19E+00	9.83E-01
Lower limit	2.40E+00	7.39E-01

Supplementary Figure 5. Carbohydrate gel analysis of intact chondroitin sulfate, using 15 % carbohydrate gel and staining with alcian blue. Lanes: Lane 1: ladder, Lane 2: dp10, Lane 3-5: CS from cells (recombinant MG1655), Lane 6: ladder

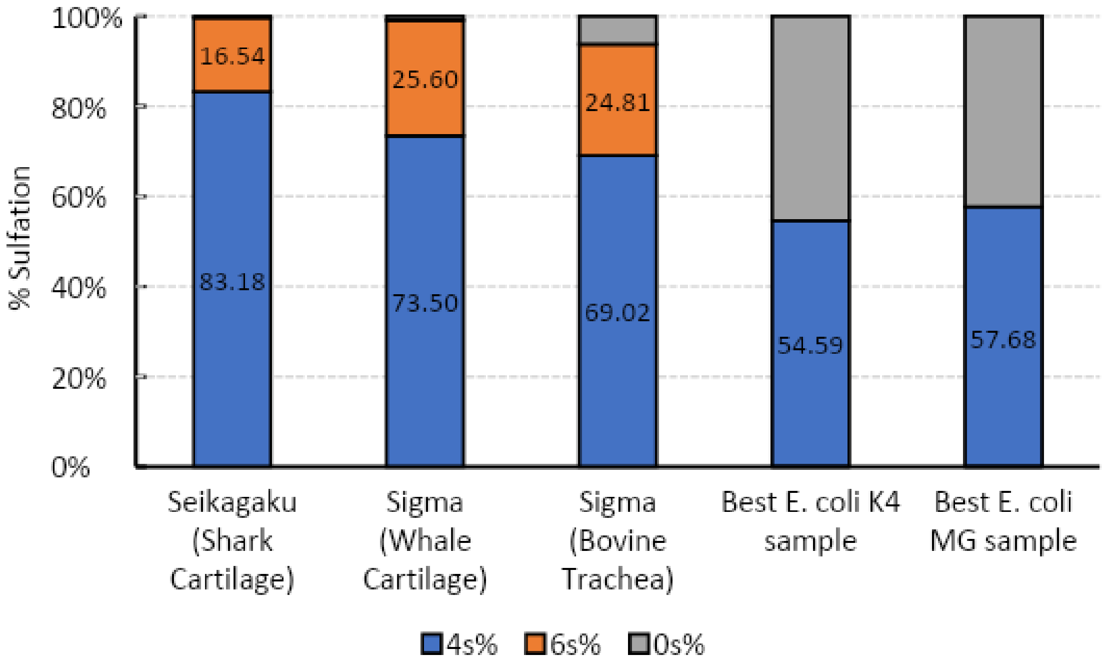

Supplementary Figure 6. Comparison between disaccharide composition in commercial animal-CS-A and microbial-CS-A obtained from shake flasks. (Xs% = % composition of X-*O*-sulfation) Source data are provided as a Source Data file.

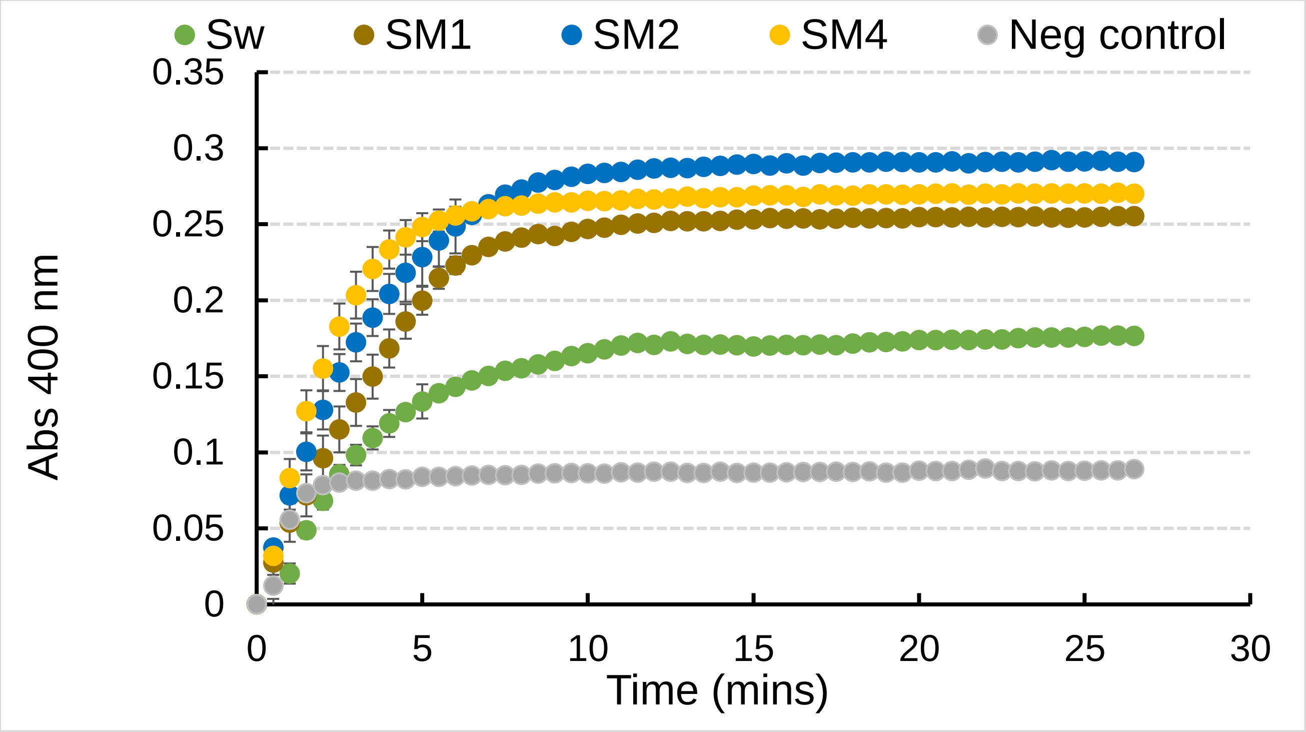

Supplementary Figure 7. Absorbance profiles of S_w and mutant activities (Neg control = no enzyme control) from aryl sulfotransferase IV colorimetric activity assay. Source data are provided as a Source Data file.

Supplementary Table 1. Secretion of chondroitin in K4 Δ*kfoE* Δ*cysH* (DE3) with and without sulfation[1].

Cell Type	Intracellular CS (μg/gDCW)	Extracellular CS (μg/L)	Intracellular CS sulfation %	Extracellular CS sulfation %	% secretion
**K4 Δ*kfoE* Δ*cysH* (DE3)**	145.64 ± 46.35	14.51 ± 6.23	0 ± 0	0 ± 0	98.96
**K4 Δ*kfoE* Δ*cysH* (DE3) S_w**	126.64 ± 15.56	10.76 ± 1.50	21.31 ± 1.35	0 ± 0	98.78
**MG1655 Δ*cysH* (DE3) PCAF**	39.24 ± 1.56	0 ± 0	0 ± 0	0 ± 0	0
**MG1655 Δ*cysH* (DE3) $PCAFS_w$**	13.14 ± 0.85	0 ± 0	57.68 ± 6.39	0 ± 0	0

[1] Values indicate average ± standard deviation.

Source data are provided as a Source Data file.

Supplementary Table 2. List of plasmids used in this study.

**Plasmids**	**Description**	**Antibiotic marker**	**Reference**
pKD46	Plasmid expressing the Red recombinase system under control of arabinose inducible promoter	Ampicillin	[1]
pKD4	Plasmid containing frt-flanked kanamycin resistance cassette.	Ampicillin, Kanamycin	[1]
pTKS/CS	Plasmid containing 25bp landing pad regions; tetracycline resistance gene and I-SceI recognition sites	Chloramphenicol, Tetracycline	[2]
pTKRED	Helper plasmid expressing λ red recombinase and yeast restriction enzyme I-SceI	Spectinomycin	[2]
pTKIP	Donor plasmid with I-SceI cut sites flanking an insertion site and 25bp landing pad	Kanamycin	[2]
pTKIP-T7RNA	pTKIP with T7 RNA polymerase gene cloned; flanked by I-Sce-I sites	Kanamycin	This Study
pETM6	ePathbrick vector with T7 promoter, lacO operator and rrnB terminator	Ampicillin	[3]
pETM6-S_w	pETM6 overexpressing S_w (chondroitin-4-*O*-sulfotransferase)	Ampicillin	[4]
pETM6-*DNCQ*S_w	pETM6 overexpressing *cysDN*, *cysC*, *cysQ* and S_w in monocistronic form	Ampicillin	This study
pETM6-*Q*S_w	pETM6 overexpressing *cysQ* and S_w in monocistronic form	Ampicillin	This study
pdCas9	Low copy number plasmid expressing dCas9 (*S. pyogenes*) and gRNA constitutively	Chloramphenicol	[5]
pdcysH	pdCas9 cloned with spacer targeting *cysH*	Chloramphenicol	[6]
pETM6-PCAF	pETM6 expressing *kfoC*, *kfoA* and *kfoF* in pseudo-operon configuration	Ampicillin	[7]
pETM6-PCAFS_w	pETM6-PCAF expressing S_w additionally in pseudo-operon configuration	Ampicillin	This study
pET32LICS_w	pET32a-LIC expressing S_w with N-terminal Trx and His tags	Ampicillin	[8]

pET32LICS$_{M1}$	pET32a-LIC expressing S_w mutant 1 with 2 PROSS-generated mutations	Ampicillin	This study
pET32LICS$_{M2}$	pET32a-LIC expressing S_w mutant 2 with 4 PROSS-generated mutations	Ampicillin	This study
pET32LICS$_{M4}$	pET32a-LIC expressing S_w mutant 4 with 12 PROSS-generated mutations	Ampicillin	This study
pdCas9_dT1	pdCas9 cloned with spacer_T1 (30bp) using golden gate cloning technique	Chloramphenicol	This study
pdCas9_dT2	pdCas9 cloned with spacer_T2 (30bp) using golden gate cloning technique	Chloramphenicol	This study
pdCas9_dT3	pdCas9 cloned with spacer_T3 (30bp) using golden gate cloning technique	Chloramphenicol	This study
pdCas9_dM1	pdCas9 cloned with spacer_M1 (30bp) using golden gate cloning technique	Chloramphenicol	This study
pdCas9_dM2	pdCas9 cloned with spacer_M2 (30bp) using golden gate cloning technique	Chloramphenicol	This study

Supplementary Table 3. List of primers used in this study.

**Primers**	**Sequence (5'-3')**
k4_dkfoE_F	TGCAATATGACCTTAGAAGAGATTTCTAATATGTTAGAACAGGAGAAAAA ACACGTCTTGAGCGATTGTG
k4_dKfoe_R	ATATCCAGCCTTGAAAAAACGCGAACTCATCCCCGCCATTGGAATTATAA ACGGCTGACATGGGAATTAG
chk_kfoE_F	GTAGACGAACACTATGATGAATTAAAAG
chk_kfoE_R	GAATAGAACCAAATTCGGCCCC
DELE_pETM_F	ATGAGTCTGAAAGAAAAAACACAATCTCTGTTTGCCAACGCATTTGGCTA ATGACCATGATTACGGATTC
DELE_pETM_R	TCAGCACTGTCCTGCTCCTTGTGATGGTTTACAAACGTAAAAAGTCTCTT CCGGCTGACATGGGAATTAG
check_T7_F	CGGGCAATGGATTCTGGTTTC
check_T7_inside	CTGTTGTTACGCAGTTGCAGATG
T7_lacUV5_F	CGCGCGGGGCCC GCTTCCGGCTCGTATAATGTGTG
T7_lacUV5_R	CGCGCG GATATC TTACGCGAACGCGAAGTCCG
K4_40UScysH_F	CGTGAGCGTCGCATCAGGCAAGGCAAACAGTGAGGAATCTgtgtaggctggagctgcttc
K4_40UScysH_R	CATCATTTCTGACAGAGGCGTTTAATTTATCCGGCAATATatgggaattagccatggtcc
delcysH_DW_seq_f	GCCCCCATCATTTCTGACAGAGGCG
delcysH_DW_seq_r	CCTGATAAGAACGCGTGAGCGTCGC
C4ST_Mut4_PCR_F	CGGAGCTCGGGATCCC
C4ST_Mut4_PCR_R	GTCGGCGATCTCGAGTTATTCC
Mut1(Glu127His)_F	GAAGTTCCTGTTTGTCCGGCATCCCTTCGAGAGGCTAGTG
Mut 1(Glu127His)_R	CACTAGCCTCTCGAAGGGATGCCGGACAAACAGGAACTTC
Mut 1(Tyr238Ser)_F	AGTGGGCAGCTCCCTGAAGTTCCCCACCTA
Mut 1(Tyr238Ser)_R	TAGGTGGGGAACTTCAGGGAGCTGCCCACT
Mut 2(Ala245Gly)_F	GAAGTTCCCCACCTATGGAAAGTCTACGAGAACTAC
Mut 2(Ala245Gly)_R	GTAGTTCTCGTAGACTTTCCATAGGTGGGGAACTTC
Mut 2(Lys117Arg)_F	GAAATCAACCACCGCTTGCGAAGCTACATGAAGTTCC
Mut 2(Lys117Arg)_R	GGAACTTCATGTAGCTTCGCAAGCGGTGGTTGATTTC
Sp_T1_1	AAACAACGCGTGAAGAGGCAATGCTGACATCATGG
Sp_T1_2	AAAACCATGATGTCAGCATTGCCTCTTCACGCGTT
Sp_T2_1	AAACCTTCACGCGTTCGGTATAACGCCAGACCGAG
Sp_T2_2	AAAACTCGGTCTGGCGTTATACCGAACGCGTGAAG
Sp_T3_1	AAACCTGGCGTGCGATATGACTTCGTCAAATTTTG
Sp_T3_2	AAAACAAAATTTGACGAAGTCATATCGCACGCCAG
Sp_M1_1	AAACCTACGGTGACTTTCTGGACTTCAAATCCACG
Sp_M1_2	AAAACGTGGATTTGAAGTCCAGAAAGTCACCGTAG
Sp_M2_1	AAACGACGGAACTTACCAAAGCGTGTTCGTATTTG
Sp_M2_2	AAAACAAATACGAACACGCTTTGGTAAGTTCCGTC

Supplementary Table 4. Sequences of wildtype and mutant sulfotransferases used in this study.

	Sequence
S_W	CAGGAACTCTACAACCCAATCCAGCTGGAGCTCTCAAACACTGCTGTCCTGCACCAG<br>ATGCGGCGGGACCAGGTGACAGACACGTGCCGAGCCAACAGCGCCACAAGCCGTA<br>AGCGGAGGGTGCTGACCCCCAACGACCTGAAGCACTTGGTGGTGGATGAGGACCA<br>CGAGCTCATCTACTGCTACGTGCCCAAGGTGGCCTGCACCAACTGGAAGCGGCTCA<br>TGATGGTCCTGACCGGGCGGGGGAAGTACAGCGACCCCATGGAGATCCCGGCCAA<br>CGAGGCACACGTCTCCGCCAACCTGAAGACCCTGAACCAGTACAGCATCCCAGAAA<br>TCAACCACCGCTTGAAAAGCTACATGAAGTTCCTGTTTGTCCGGGAGCCCTTCGAGA<br>GGCTAGTGTCCGCCTACCGCAACAAGTTCACCCAGAAGTACAACATCTCCTTCCACA<br>AGCGGTACGGCACCAAGATCATCAAACGCCAGCGGAAGAACGCCACCCAGGAGGC<br>CCTGCGCAAAGGGGACGATGTCAAATTCGAGGAGTTTGTGGCCTATCTCATCGACCC<br>ACACACCCAGCGGGAGGAGCCTTTCAACGAACACTGGCAAACCGTCTACTCACTCT<br>GCCATCCCTGCCACATCCACTATGACCTCGTGGGCAAGTACGAGACACTGGAAGAG<br>GATTCTAATTACGTCCTGCAGCTGGCAGGAGTGGGCAGCTACCTGAAGTTCCCCACC<br>TATGCAAAGTCTACGAGAACTACTGATGAAATGACCACAGAATTCTTCCAGAACATCA<br>GCTCAGAGCACCAAACGCAGCTGTACGAAGTCTACAAACTCGATTTTTTAATGTTCAA<br>TTACTCAGTGCCAAGCTACCTGAAATTGGAATAA
S_{M1}	CAGGAACTGTATAACCCGATTCAGCTGGAACTGAGCAACACCGCGGTGCTGCATCAG<br>ATGCGCCGCGATCAGGTGACCGATACCTGCCGCGCGAACAGCGCGACCAGCCGC<br>AAACGCCGCGTGCTGACCCCGAACGATCTGAAACATCTGGTGGTGGATGAAGATC<br>ATGAACTGATTTATTGCTATGTGCCGAAAGTGGCGTGCACCAACTGGAAACGCCTG<br>ATGATGGTGCTGACCGGCCGCGGCAAATATAGCGATCCGATGGAAATTCCGGCGA<br>ACGAAGCGCATGTGAGCGCGAACCTGAAAACCCTGAACCAGTATAGCATTCCGGA<br>AATTAACCATCGCCTGAAAAGCTATATGAAATTTCTGTTTGTGCGCCATCCGTTTGA<br>ACGCCTGGTGAGCGCGTATCGCAACAAATTTACCCAGAAATATAACATTAGCTTTC<br>ATAAACGCTATGGCACCAAAATTATTAAACGCCAGCGCAAAAACGCGACCCAGGAA<br>GCGCTGCGCAAAGGCGATGATGTGAAATTTGAAGAATTTGTGGCGTATCTGATTGA<br>TCCGCATACCCAGCGCGAAGAACCGTTTAACGAACATTGGCAGACCGTGTATAGC<br>CTGTGCCATCCGTGCCATATTCATTATGATCTGGTGGGCAAATATGAAACCCTGGA<br>AGAAGATAGCAACTATGTGCTGCAGCTGGCGGGCGTGGGCAGCAGCCTGAAATTT<br>CCGACCTATGCGAAAAGCACCCGCACCACCGATGAAATGACCACCGAATTTTTCA<br>GAACATTAGCAGCGAACATCAGACCCAGCTGTATGAAGTGTATAAACTGGATTTTC<br>TGATGTTTAACTATAGCGTGCCGAGCTATCTGAAACTGGAA
S_{M2}	CAGGAACTGTATAACCCGATTCAGCTGGAACTGAGCAACACCGCGGTGCTGCATCAG<br>ATGCGCCGCGATCAGGTGACCGATACCTGCCGCGCGAACAGCGCGACCAGCCGC<br>AAACGCCGCGTGCTGACCCCGAACGATCTGAAACATCTGGTGGTGGATGAAGATC<br>ATGAACTGATTTATTGCTATGTGCCGAAAGTGGCGTGCACCAACTGGAAACGCCTG<br>ATGATGGTGCTGACCGGCCGCGGCAAATATAGCGATCCGATGGAAATTCCGGCGA<br>ACGAAGCGCATGTGAGCGCGAACCTGAAAACCCTGAACCAGTATAGCATTCCGGA<br>AATTAACCATCGCCTGCGCAGCTATATGAAATTTCTGTTTGTGCGCCATCCGTTTGA<br>ACGCCTGGTGAGCGCGTATCGCAACAAATTTACCCAGAAATATAACATTAGCTTTC<br>ATAAACGCTATGGCACCAAAATTATTAAACGCCAGCGCAAAAACGCGACCCAGGAA<br>GCGCTGCGCAAAGGCGATGATGTGAAATTTGAAGAATTTGTGGCGTATCTGATTGA

	TCCGCATACCCAGCGCGAAGAACCGTTTAACGAACATTGGCAGACCGTGTATAGC<br>CTGTGCCATCCGTGCCATATTCATTATGATCTGGTGGGCAAATATGAAACCCTGGA<br>AGAAGATAGCAACTATGTGCTGCAGCTGGCGGGCGTGGGCAGCAGCCTGAAATTT<br>CCGACCTATGGCAAAAGCACCCGCACCACCGATGAAATGACCACCGAATTTTTTCA<br>GAACATTAGCAGCGAACATCAGACCCAGCTGTATGAAGTGTATAAACTGGATTTTC<br>TGATGTTTAACTATAGCGTGCCGAGCTATCTGAAACTGGAA
S_{M4}	CAGGAACTCTACAACCCATCCCAGCTGGAGCTCTCAAACACTGCTGTCCTGCACCAG<br>ATGCGGCGGGACCAGGTGACAGACACGTGCCAAGCCAACAGCGCCACAAGCCGT<br>AAGCGGAGGGTGCTGACCCCCAACGACCTGAAGCACTTGGTGGTGGATGAGGAC<br>CACGAGCTCATCTACTGCTACGTGCCCAAGGTGGCCTGCACCAACTGGAAGCGGC<br>TCATGATGGTCCTGACCGGGCGGGGGAAGTACAGCGACCCCATGGAGATCCCGG<br>CCAACGAGGCACACGTCTCCGCCAACCTGAAGACCCTGAACGAGTACAGCATCCC<br>AGAAATCAACCACCGCTTGCGAAACTACATGAAGTTCCTGTTTGTCCGGCATCCCT<br>TCGAGAGGCTAGTGTCCGCCTACCGCAACAAGTTCACCCAGAAGTACAACACCTC<br>CTTCCACAAGCGGTACGGCACCAAGATCATCAAACGCCAGCGGAAGAACGCCACC<br>CAGGAGGCCCTGCGCAAAGGGGACGATGTCAAATTCGAGGAGTTTGTGGCCTATC<br>TCATCGACCCACACACCCAGCGGGAGGAGCCTTTCAACGAACACTGGCAAACCGT<br>CTACTCACTCTGCCATCCCTGCCACATCCACTATGACCTCGTGGGCAAGTACGAGA<br>CACTGGAAGAGGATGCTAATTACGTCCTGCAGCTGGCAGGAGTGGGCGACTCCCT<br>GAAGTTCCCCACCTATGGAAAGTCTACGAGAACTACTGATGAAATGACCACAGAAT<br>TCTTCCAGAACATCAGCTCAGAGCACCAAACGCAGCTGTACCAAGTCTACAAACTC<br>GATTTTTTAATGTTCAATTACTCAGTGCCAAGCTACCTGAAATTGGAATAA

Supplementary References

1. Datsenko, K. A. & Wanner, B. L. One-step inactivation of chromosomal genes in Escherichia coli K-12 using PCR products. *Proceedings of the National Academy of Sciences* **97**, 6640–6645 (2000).
2. Kuhlman, T. E. & Cox, E. C. Site-specific chromosomal integration of large synthetic constructs. *Nucleic Acids Research* **38**, e92–e92 (2010).
3. Xu, P., Vansiri, A., Bhan, N. & Koffas, M. A. G. ePathBrick: a synthetic biology platform for engineering mMetabolic pathways in *E. coli*. *ACS Synth. Biol.* **1**, 256–266 (2012).
4. He, W. Metabolic engineering and applied enzymology for the preparation of nutraceutical/pharmaceutical chondroitin sulfate. (Rensselaer Polytechnic Institute, 2017).
5. Bikard, D. *et al.* Programmable repression and activation of bacterial gene expression using an engineered CRISPR-Cas system. *Nucleic Acids Research* **41**, 7429–7437 (2013).
6. Badri, A., Williams, A., Xia, K., Linhardt, R. J. & Koffas, M. A. G. Increased 3′‐phosphoadenosine‐5′‐phosphosulfate levels in engineered *Escherichia coli* cell lysate facilitate the *in vitro* synthesis of chondroitin sulfate A. *Biotechnol. J.* **14**, 1800436 (2019).

7. He, W. *et al.* Production of chondroitin in metabolically engineered E. coli. *Metabolic Engineering* **27**, 92–100 (2015).
8. He, W. *et al.* Expression of chondroitin-4-*O*-sulfotransferase in *Escherichia coli* and *Pichia pastoris*. *Appl Microbiol Biotechnol* **101**, 6919–6928 (2017).
