# Supplementary material for: Complete biosynthesis of a sulfated chondroitin in Escherichia coli
Source: Nat Commun. 2021 Mar 2;12:1389. doi: 10.1038/s41467-021-21692-5 (PMC7925653; doi:10.1038/s41467-021-21692-5)
Supplement: Supplementary file 2 — Peer Review [file 41467_2021_21692_MOESM2_ESM.pdf]

Reviewer #1 (Remarks to the Author):

In this manuscript, the authors attempted to develop a metabolically engineered E. coli strain for the complete biosynthesis of animal-free CS. During the course of this study, the authors identified several important factors that control CS sulfation level in E. coli, achieving an efficient intracellular CS production. All data presented in this manuscript are invaluable in terms of the practical realization of the animal-free production of sulfated GAGs.

Major points #1
Considering the data described in this manuscript, some experimental designs seem not to be the best to assess the CS production level in the engineered E. coli.
For example, although the authors found that a mutant chondroitin 4-O-sulfotransferase SM2 is the most efficient enzyme that produced CS with a three-fold increased sulfation compared with wildtype Sw, the following experiments were conducted only under the control of wildtype Sw, but not of its mutant SM2. If the lower intracellular sulfation levels in the E. coli (Fig. S3) are attributable to the usage of pET32LIC for sulfotransferase expression as described in the text (page 12, line 12 to page 13, line 7), further experiments should be performed using E. coli, where mutant enzymes such as SM2 are expressed based on pETM6 vector, instead of pET32LIC.
In addition, the authors described that E. coli MG1655ΔcysH(DE3) with pETM6-PCAFSw (without modifying any transporter expression) led to a high intracellular CS sulfation level of 58% (Fig. 3E). Although its CS titer was markedly decreased compared with that in the parental MG1655ΔcysH(DE3), this strain also seems to be one of useful platform for achievement of animal-free CS production. Unlike K4 strains, why can this strain produce CS with higher sulfation level? The authors should show the related (additional) evidence and/or discuss this point.
Despite the title of this manuscript "Complete biosynthesis of a sulfated glycosaminoglycan in Escherichia coli", the authors only optimized metabolic systems in E. coli that are required for efficient CS-A production. Are these optimized conditions applicable for the animal-free synthesis of the other types of sulfated GAGs, such as CS-C, and/or heparan sulfates? To address this question, additional experiments using vector constructs carrying the other GAG sulfotransferase genes (chondroitin 6-O-sulfotransferase etc.) should be done. Otherwise, the manuscript title should be reconsidered.

Major points #2
To evaluate whether the E. coli-derived CS-A is functionally equivalent to the animal-derived ones, its structural and biological information is the most essential. In terms of structural information, at least average molecular weight of the E. coli-derived CS preparations should be examined. And also, the authors should compare the immunogenic properties and/or biological activities of the purified bacterial CS-A with those of animal-derived CS-A.

Minor points #1
Page 4, line 7, a citation of Fig. 1 in the text should be replaced with Fig. 1(B). Accompanied with this correction, Fig. 1(A) is not cited in the text. And also, alphabetic characters "A" and "B" should be added in Fig. 1 (Page 5).

Minor points #2
Page 5, Fig. 1, the chemical structure of CS-E is mistaken.

Minor points #3
Page 10, line 7, a citation of Fig. 3(C) in the text should be replaced with Fig. 3(D). Accompanied with this correction, Fig. 3(C) is not cited in the text.

Minor points #4
Page 21, line 25, a description "chondroitinase" should be replaced with "chondroitin".

Reviewer #2 (Remarks to the Author):

The authors engineered E. coli to produce CS. This required the engineering of chondroitin and PAPS biosynthesis as well as expression of a sulfotransferase. They achieved one-step microbial production of a sulfated GAG at a titer of approx14 μg/g. This is an interesting study and a classic example of metabolic pathway engineering in E. coli.

Results:

The section entitled "Assembly of metabolic pathways for microbial CS production" should be reduced to a few sentences and folded into the next section as it contains no results but mostly introduction and methods

- The first 2 paragraph describing CS biosynthesis (lines 97 to 118) should be moved to the introduction.
- Lines 119-121, the sentence "…..of more pro-CS components needed for CS biosynthesis in E. coli K4, when compared to other E. coli strains (Fig. 2(B)), E. coli K4 was selected to assemble the required elements for CS biosynthesis" is not very clear. More precise would be "Given that E. coli K4 harbors a UDP-N-acetylglucosamine-/UDP glucosamine-4-epimerase and chondroitin synthase, it was selected over E. coli MG1655 for assembly of a CS biosynthesis pathway.
- Fig 2 (B) can be eliminated as is brings nothing additional to the suggested text.
- Phenotype of kfoE deletion known.

PAPS accumulation facilitates intracellular CS synthesis

- Lines 138-139 "……and previously demonstrated successful Sw expression,…..", this data needs to be shown here or cited if from a previous study.
- Lines 140-142: "Although the PAPS biosynthetic pathway is present in most cells, it is not always highly active and does not result in accumulation of sufficient PAPS needed for the preparation of high-yield sulfated products". This sentence is very imprecise. What is most? Or not always highly active? It also needs a citation.
- It is not clear as to why both a repression and deletion strategy was used for cysH? What is the phenotype/fitness of the delta cysH?
- Fig 3: (A) what does the dash arrow represent? Ps and Ss are missing. Color scheme is confusing. Sulfation% is blue in (B) but green in (E). How is % sulfation calculated? Seems to me that combining Fig 3 (B) and (D) would allow a simple comparison. Same could be said for (B) and (E). Could/should eliminate (C)
- Line 168-170 "This could be the result of an increased metabolic burden on the cell or the accumulation of toxic intermediates in the PAPS biosynthetic pathway." What is the cell fitness/growth rates/yields for the various strains? This would give a good indication of potential burden/toxicity.
- What is the Sw or PAPS levels in the two strains reported in Fig 3 (E)? Could this data help clarify the metabolic burden statement on line 178-179?

Improving sulfotransferase activity enhances CS sulfation

- Line 181-182: "Having explored fundamental PAPS pathways to obtain improved CS sulfation, we sought to improve the production of active Sw required for CS synthesis." But the data in the previous section seems to point to some other factor (metabolic burden?) limiting CS synthesis?
- Line 185-187: "Based on the hypothesis that increased solubility and stability of Sw would improve CS sulfation levels, we relied on a computational approach to identify mutations enhancing these properties." Some data suggesting that Sw solubility and stability is an issue would better justify this section. Or at least a comparison between the wt and variants. Without that information this section becomes a "shot in the dark" that did yield some benefits (as shown in Fig 4.) but cannot be explained or reproduced for another protein/system.
- Description of the variants is somewhat confusing. Line 199-200 reports 10 potential mutations (although 12 are shown in Fig S2B) that could improve protein solubility and stability yet only 3 variants are reported (SM1, SM2 and SM4) each containing a subset of the 10/12 mutations (2 for

SM1, 4 for SM2 and 12 for SM4). How/why were these three protein variants selected?
• Line 218-221: "The disparity between the in vitro and in vivo activities could be attributed to differences in the levels of expressed chondroitin-4-O-sulfotransferase and/or the chondroitin and PAPS substrates in the respective experiments." This is not clear. Shouldn't the chondroitin and PAPS substrates be consistent between these experiments given that the strains are isogenic with the exception of the Sw? Why not measure these substrates or Sw expression by western given that they are tagged? Would be a good idea to repeat the experiments with the Sw variants without the tags (with pETM6) to compare the data to Fig 3.

Sulfation level is dependent on fermentation conditions
• These are basic optimization experiments that probably don't warrant their own section and could be summarized in a few sentences.
• For the temperature experiments, was the shift from 37 to 16 or 20 done by air cooling? If so for this means that it probably took a while (depending on volume, shaking etc) for the media to cool from 37 to 16 resulting on protein expression and activity to occur at a temperature somewhere between 37 and the desired temperature.

Impact of cellular export mechanism on CS sulfation
• Why do three of the 5 spacers tested give higher secretion rates for CS (Fig S4)?
• Why use dCAS9 repression as opposed to a knock out, which would be much cleaner?
• Is the Sw induced in Fig 6 panel (B)?
• Fig S4 reports Relative Extracellular CS (%), Fig 6 (B) reports Relative Extracellular GAGs and Fig 6 (C) reports Intracellular CS titer (ug/gDCW). Why GAGs vs CS? It would be preferable to show all of the absolute data for these three experiments (intracellular and extracellular CS titer (ug/DCW)).

Discussion:

A discussion needs to compare/contrast/explain the results obtained here to the existing literature yet I could only find three citations and no reference to the data figures.

Reviewer #3 (Remarks to the Author):

The manuscript describes the development of engineered E. coli strains (K4 and K12 MG1655) for the production of chondroitin sulfate containing 4-O-sulfation. Various strategies used to achieve the goal include: deleting fructosyltransferase-encoding gene from E. coli K4, adding T7 RNA polymerase gene to its genome, transforming a plasmid containing chondroitin 4-O-sulfotransferase (SW), and repressing the expression of PAPS reductase (CysH). To improve the sulfation level, several additional strategies have been used. Overexpression of CysDNCQ involved in the PAPS biosynthesis decreased the level of chondroitin sulfation. The use of engineered E. coli K12 MG1655 strain with a higher level of PAPS production and a plasmid containing E. coli K4 genes involved in chondroitin synthesis and the plasmid containing Sw improved the level of CS accumulated intracellularly. Mutants of SW were designed and SM2 was shown to improve % sulfation up to three-fold. The influence of sulfation level by fermentation conditions and repression of chondroitin transporter gene was also demonstrated. It is significant that the E. coli expression systems for chondroitin sulfate production were successfully achieved. The current study, however, has not reached the level of enthusiasm needed to support the publication in Nat. Commun.
1. The current study is somewhat narrow as it focuses on O-sulfation at a single site (4-O-sulfation) without the consideration of 6-O-sulfation which is also required for the formation of chondroitin sulfate. In addition, both the expression and the sulfation levels achieved in the current study are quite low. Especially, homogeneous sulfation has not been obtained.
2. The sizes and the size distributions of the polysaccharide products are not investigated. These are important factors to consider for chondroitin sulfate production.

3. A more rigorous product characterization process (e.g. purity, NMR results) is needed.
4. Detailed comparison of expression levels and activities of different enzymes under various conditions are lacking.
Some more specific suggestions are shown below:
5. As only CS-A is produced in the report, it is not necessary to include details for CS-B, CS-C, CS-D and CS-E in Fig. 1. In addition, IdoA-containing structure CS-B belongs to dermatan sulfate, not chondroitin sulfate any more.
6. Specific mutations in SM1, SM2, and SM4 should be listed in the main manuscript.
7. More detailed biochemical characterization (expression level, kinetics, etc.) of SW and mutants (SM1, SM2, and SM4) and related results should be shown in the main manuscript or in the Supporting Information file.
8. Table S1 should be expanded and moved to the main manuscript. It should include results from the construct with 58% level of CS sulfation, and from different constructs under different conditions investigated in this study.

REVIEWER COMMENTS

Reviewer #1 (Remarks to the Author):

In this manuscript, the authors attempted to develop a metabolically engineered E. coli strain for the complete biosynthesis of animal-free CS. During the course of this study, the authors identified several important factors that control CS sulfation level in E. coli, achieving an efficient intracellular CS production. All data presented in this manuscript are invaluable in terms of the practical realization of the animal-free production of sulfated GAGs.

Considering the data described in this manuscript, some experimental designs seem not to be the best to assess the CS production level in the engineered E. coli.

For example, although the authors found that a mutant chondroitin 4-O-sulfotransferase SM2 is the most efficient enzyme that produced CS with a three-fold increased sulfating compared with wildtype Sw, the following experiments were conducted only under the control of wildtype Sw, but not of its mutant SM2.

If the lower intracellular sulfating levels in the E. coli (Fig. S3) are attributable to the usage of pET32LIC for sulfotransferase expression as described in the text (page 12, line 12 to page 13, line 7), further experiments should be performed using E. coli, where mutant enzymes such as SM2 are expressed based on pETM6 vector, instead of pET32LIC.

In addition, the authors described that E. coli MG1655ΔcysH(DE3) with pETM6-PCAFSw (without modifying any transporter expression) led to a high intracellular CS sulfation level of 58% (Fig. 3E). Although its CS titer was markedly decreased compared with that in the parental MG1655ΔcysH(DE3), this strain also seems to be one of useful platform for achievement of animal-free CS production.

Response to Comment 1: We thank you for this observation. We explored all the four aspects to improve sulfation in parallel and that is why the results of one section had not been transferred to the others. However, in response to this comment, we have now constructed and characterized strains combining all the successful strategies observed from the four aspects tested. These strains had significantly lower sulfation levels suggesting the requirement for some optimization. We have included the new experimental data in the manuscript. In addition the title of the manuscript has been modified to better reflect the true nature of our work.

Reviewer 1 Comment 2:
Unlike K4 strains, why can this strain produce CS with higher sulfation level? The authors should show the related (additional) evidence and/or discuss this point.

Response to Comment 2: Our MG strain was able to make CS with higher sulfation level because it accumulated higher PAPS. We observe that different strains respond to cysH deletion differently and thus accumulate PAPS at different levels.

Reviewer 1 Comment 3:
Despite the title of this manuscript “Complete biosynthesis of a sulfated glycosaminoglycan in Escherichia coli”, the authors only optimized metabolic systems in *E. coli* that are required for efficient CS-A production.
Are these optimized conditions applicable for the animal-free synthesis of the other types of sulfated GAGs, such as CS-C, and/or heparan sulfates? To address this question, additional experiments using vector constructs carrying the other GAG sulfotransferase genes (chondroitin 6-O-sulfotransferase etc.) should be done. Otherwise, the manuscript title should be reconsidered.

Response to Comment 3: In response to this comment, we have now re-titled our manuscript to reflect that only chondroitin sulfate has been biosynthesized. While making more types of chondroitin sulfates is beyond the scope of this manuscript, we believe that the transferability of this study to other CS types depends primarily on the successful expression of the corresponding sulfotransferases. We have also updated the discussion section to include a few sentences on this point.

Reviewer 1 Comment 4:
To evaluate whether the E. coli-derived CS-A is functionally equivalent to the animal-derived ones, its structural and biological information is the most essential. In terms of structural information, at least average molecular weight of the E. coli-derived CS preparations should be examined. And also, the authors should compare the immunogenic properties and/or biological activities of the purified bacterial CS-A with those of animal-derived CS-A.

Response to Comment 4: We thank the reviewer for this suggestion. We have now performed molecular weight analysis on CS-A produced from our best strain and added it to the results section in the manuscript. Method used for determining molecular weight has also been added to the method section. Assessing the immunogenic properties of our bacterial CS-A requires further scale-up, purification and cell-culture work that are beyond the scope of this manuscript. However, they are currently on-going and will be part of a separate follow-up manuscript.

Reviewer 1 Comment 5:
Page 4, line 7, a citation of Fig. 1 in the text should be replaced with Fig. 1(B). Accompanied with this correction, Fig. 1(A) is not cited in the text. And also, alphabetic characters “A” and “B” should be added in Fig. 1 (Page 5). Page 5, Fig. 1, the chemical structure of CS-E is mistaken.

Response to Comment 5: Thank you for pointing this out. We have included the characters and updated the in-text citation. We have also corrected the error in the structure of CS-E.

Reviewer 1 Comment 6:
Page 10, line 7, a citation of Fig. 3(C) in the text should be replaced with Fig. 3(D). Accompanied with this correction, Fig. 3(C) is not cited in the text.

Response to Comment 6: Thank you for pointing this out. We corrected this citation and included a citation for 3(C).

Reviewer 1 Comment 7:
Page 21, line 25, a description “chondroitinase” should be replaced with “chondroitin”

Response to Comment 7: The above line in the manuscript has been corrected accordingly.

Reviewer #2 (Remarks to the Author):

The authors engineered E. coli to produce CS. This required the engineering of chondroitin and PAPS biosynthesis as well as expression of a sulfotransferase. They achieved one-step microbial production of a sulfated GAG at a titer of approx14 µg/g. This is an interesting study and a classic example of metabolic pathway engineering in E. coli.

Reviewer 2 Comment 1:
The section entitled “Assembly of metabolic pathways for microbial CS production” should be reduced to a few sentences and folded into the next section as it contains no results but mostly introduction and methods
• The first 2 paragraph describing CS biosynthesis (lines 97 to 118) should be moved to the introduction.
• Lines 119-121, the sentence “…..of more pro-CS components needed for CS biosynthesis in E. coli K4, when compared to other E. coli strains (Fig. 2(B)), E. coli K4 was selected to assemble the required elements for CS biosynthesis” is not very clear. More precise would be “Given that E. coli K4 harbors a UDP-N-acetylglucosamine-/UDP glucosamine-4-epimerase and chondroitin synthase, it was selected over E. coli MG1655 for assembly of a CS biosynthesis pathway.
• Fig 2 (B) can be eliminated as is brings nothing additional to the suggested text.
• Phenotype of kfoE deletion known.

Response to Comment 1: Thank you for this suggestion. In response to this, we have reduced the section to a few sentences, removed fig 1(B) and moved some parts to the introduction.

Reviewer 2 Comment 2:
PAPS accumulation facilitates intracellular CS synthesis
• Lines 138-139 “……and previously demonstrated successful Sw expression,…..”, this data needs to be shown here or cited if from a previous study.

Response to Comment 2: Thank you for pointing this out. The appropriate reference has been included in this sentence.

Reviewer 2 Comment 3:

• Lines 140-142: “Although the PAPS biosynthetic pathway is present in most cells, it is not always highly active and does not result in accumulation of sufficient PAPS needed for the preparation of high-yield sulfated products”. This sentence is very imprecise. What is most? Or not always highly active? It also needs a citation.

Response to Comment 3: In response to this comment, we have removed this sentence from the manuscript.

Reviewer 2 Comment 4:

• It is not clear as to why both a repression and deletion strategy was used for cysH? What is the phenotype/fitness of the delta cysH?

Response to Comment 4: Thank you for this comment. The deletion strain is stable, but we have not done any specific fitness tests. However, growth is definitely poorer (3 OD (Del) compared to 24 OD (WT)) so we tested the repression to see if overall titers were higher without compromising on growth. Since that was not the case, we didn’t do any further analysis with that strain.

Reviewer 2 Comment 5:

• Fig 3: (A) what does the dash arrow represent? Ps and Ss are missing. Color scheme is confusing. Sulfation% is blue in (B) but green in (E). How is % sulfation calculated? Seems to me that combining Fig 3 (B) and (D) would allow a simple comparison. Same could be said for (B) and (E). Could/should eliminate (C)

• Line 168-170 “This could be the result of an increased metabolic burden on the cell or the accumulation of toxic intermediates in the PAPS biosynthetic pathway.” What is the cell fitness/growth rates/yields for the various strains? This would give a good indication of potential burden/toxicity.

Response to Comment 5: In response to this comment we have eliminated 3(C) and included the phosphates and sulfates to complete the represented pathway. We have also corrected the color scheme. However, since 3(B) shows strains that are based on the K4 strain and 3(E) (now 3D) shows MG1655 strains and 3(D) (now 3C) describes a different aspect of our study, we would hence like to keep them as separate panels. Additionally, in response to the second part of the comment, we have removed the ambiguous sentence since it was more of a speculation without enough backing.

Reviewer 2 Comment 6:

• What is the Sw or PAPS levels in the two strains reported in Fig 3 (E)? Could this data help clarify the metabolic burden statement on line 178-179?

Response to Comment 6: Thank you. In response to this comment, we have quantified PAPS levels in the MG1655ΔcysH(DE3) pETM6-PCAFS$_w$ and included the data in the figure. We performed an SDS PAGE analysis to check expression levels (shown below). However, since the results of this study were inconclusive, we have not included these in our revised manuscript.

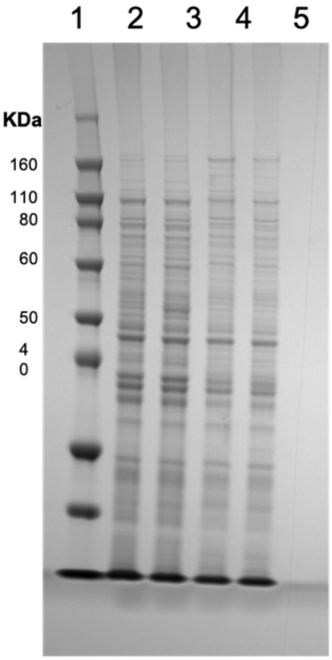

	Key
Lane 1	*Ladder*
Lane 2	*MG1655cysH(DE3) pETM6-PCAFS$_W$*
Lane 3	*MG1655cysH(DE3) pETM6-PCAFS$_{M2}$*
Lane 4	k4kfoEcysH(DE3) pETM6-S$_W$ pdcas9-dkpsM1
Lane 5	k4kfoEcysH(DE3) pETM6-S$_{M2}$ pdcas9-dkpsM1

Sulfotransferase size: ~ 50 kDa

Reviewer 2 Comment 7:
Improving sulfotransferase activity enhances CS sulfation
• Line 181-182: "Having explored fundamental PAPS pathways to obtain improved CS sulfation, we sought to improve the production of active Sw required for CS synthesis." But the data in the previous section seems to point to some other factor (metabolic burden?) limiting CS synthesis?
Response to Comment 7: In response to this comment, we have removed these sentences from the manuscript.

Reviewer 2 Comment 8:
• Line 185-187: "Based on the hypothesis that increased solubility and stability of Sw would improve CS sulfation levels, we relied on a computational approach to identify mutations enhancing these properties." Some data suggesting that Sw solubility and stability is an issue would better justify this section. Or at least a comparison between the wt and variants. Without that information this section becomes a "shot in the dark" that did yield some benefits (as shown in Fig 4.) but cannot be explained or reproduced for another protein/system.

Response to Comment 8: Thank you for this comment. This was a shot-in-the-dark attempt. PROSS is a server that predicts variants with possibly improved solubility and stability. While the mutants showed improved activity, unfortunately, we could not see any solubility or stability improvements in them. In order to remove the ambiguity associated with this, we have changed our description of this section and the server.

Reviewer 2 Comment 9:
• Description of the variants is somewhat confusing. Line 199-200 reports 10 potential mutations (although 12 are shown in Fig S2B) that could improve protein solubility and

stability yet only 3 variants are reported (SM1, SM2 and SM4) each containing a subset of the 10/12 mutations (2 for SM1, 4 for SM2 and 12 for SM4).
How/why were these three protein variants selected?

Response to Comment 9: The three variants tested and reported were predicted by the web-based tool, PROSS. Upon inspection, these three variants were found to contain the mutations mentioned above.

Reviewer 2 Comment 10:
• Line 218-221: "The disparity between the in vitro and in vivo activities could be attributed to differences in the levels of expressed chondroitin-4-O-sulfotransferase and/or the chondroitin and PAPS substrates in the respective experiments." This is not clear.
Shouldn't the chondroitin and PAPS substrates be consistent between these experiments given that the strains are isogenic with the exception of the Sw?

Response to Comment 10: The reasoning given above was for the difference between in vitro and corresponding in vivo values (S_w(in vitro) vs S_w(in vivo); S_{M1}(in vitro) vs S_{M1}(in vivo) etc.) and not between the different mutants. In response to this comment, we have explicitly clarified and rephrased this in the text.

Reviewer 2 Comment 11:
Why not measure these substrates or Sw expression by western given that they are tagged?

Response to Comment 11: While our BL21(DE3) strains expressed tagged enzyme, our final CS production strains did not have tags. However, we have now included an SDS-PAGE in the supplementary section.

Reviewer 2 Comment 12:
Would be a good idea to repeat the experiments with the Sw variants without the tags (with pETM6) to compare the data to Fig 3.

Response to Comment 12: We have done and reported this for the best performing mutant, S_{M2}, in the supplementary section. In response to this comment we have moved it to the main manuscript.

Reviewer 2 Comment 13:
Sulfation level is dependent on fermentation conditions
• These are basic optimization experiments that probably don't warrant their own section and could be summarized in a few sentences.

Response to Comment 13: Thank you for this suggestion. We have cut down this section to one paragraph. However, we have retained it as a separate section to demarcate it and avoid confusion for the reader with other sections.

Reviewer 2 Comment 14:
• For the temperature experiments, was the shift from 37 to 16 or 20 done by air cooling? If so for this means that it probably took a while (depending on volume, shaking etc) for the media to cool from 37 to 16 resulting on protein expression and activity to occur at a temperature somewhere between 37 and the desired temperature.

Response to Comment 14: Thank you for the question. It took less than 15 mins for the temperature drop. Meanwhile, the flasks were cooled down to RT and induced with IPTG. The flasks were only returned to the incubator after the final set temperature was reached. Based on our experimental design, we don't think this had a significant effect on our results.

Reviewer 2 Comment 15:
Impact of cellular export mechanism on CS sulfation
• Why do three of the 5 spacers tested give higher secretion rates for CS (Fig S4)?
• Why use dCAS9 repression as opposed to a knock out, which would be much cleaner?

Response to Comment 15: In this study, we were simply evaluating if sulfation was related to export of CS. While we obtained CS in the lysate, an intracellular product is not our design goal looking forward, due to its limitations as we scale-up. So, we did not want to completely eliminate export. Hence, for purposes of this study, we evaluated effect of export on sulfation by partial repression achieved by using our spacer dM_1. *Regarding the comment about some of the tested spacers having increased export – two of these three did not have significantly higher secretion rates. We do not know why dM2 showed higher export. Since this wasn't a characteristic we were looking for, we did not look into it further.*

Reviewer 2 Comment 17:
• Is the Sw induced in Fig 6 panel (B)?

Response to Comment 17: Yes, S_w *was induced in this condition. We have now specified this in the figure and legend.*

Reviewer 2 Comment 18:
• Fig S4 reports Relative Extracellular CS (%), Fig 6 (B) reports Relative Extracellular GAGs and Fig 6 (C) reports Intracellular CS titer (ug/gDCW). Why GAGs vs CS? It would be preferable to show all of the absolute data for these three experiments (intracellular and extracellular CS titer (ug/DCW)).

Response to Comment 18: In response to this suggestion, we have re-calculated from the raw data to ensure that all titers are absolute. However, due to the significantly higher titer, we are reporting extracellular CS in mg/gDCW and intracellular CS in □g/gDCW (now Fig. 5(B)).

Reviewer 2 Comment 19:
Discussion: A discussion needs to compare/contrast/explain the results obtained here to the existing literature yet I could only find three citations and no reference to the data figures.

Response to Comment 19: Thank you for this comment. We have now re-written our discussion and included references in accordance to this suggestion.

Reviewer #3 (Remarks to the Author):

The manuscript describes the development of engineered E. coli strains (K4 and K12 MG1655) for the production of chondroitin sulfate containing 4-O-sulfation. Various strategies used to achieve the goal include: deleting fructosyltransferase-encoding gene from E. coli K4, adding T7 RNA polymerase gene to its genome, transforming a plasmid containing chondroitin 4-O-sulfotransferase (SW), and repressing the expression of PAPS reductase (CysH). To improve the sulfation level, several additional strategies have been used. Overexpression of CysDNCQ involved in the PAPS biosynthesis decreased the level of chondroitin sulfation. The use of engineered E. coli K12 MG1655 strain with a higher level of PAPS production and a plasmid containing E. coli K4 genes involved in chondroitin synthesis and the plasmid containing Sw improved the level of CS accumulated intracellularly. Mutants of SW were designed and SM2 was shown to improve % sulfation up to three-fold. The influence of sulfation level by fermentation conditions and repression of chondroitin transporter gene was also demonstrated. It is significant that the E. coli expression systems for chondroitin sulfate production were successfully achieved. The current study, however, has not reached the level of enthusiasm needed to support the publication in Nat. Commun.

Reviewer 3 Comment 1:
1. The current study is somewhat narrow as it focuses on O-sulfation at a single site (4-O-sulfation) without the consideration of 6-O-sulfation which is also required for the formation of chondroitin sulfate.
In addition, both the expression and the sulfation levels achieved in the current study are quite low. Especially, homogeneous sulfation has not been obtained.

Response to Comment 1: We thank you for this comment. Our sulfation levels were comparable to animal-sourced CS-A as described in Pg. 19. We have also included additional results with further improved sulfation levels. While some types of chondroitin sulfate are sulfated at other positions, this manuscript focuses on CS-A, which is predominantly sulfated at the 4-position. We believe our approach could be extended to other types, but the work to produce these as well as to improve yields is currently underway and beyond the scope of this manuscript.

Reviewer 3 Comment 2:

2. The sizes and the size distributions of the polysaccharide products are not investigated. These are important factors to consider for chondroitin sulfate production.

Response to Comment 2: In response to this comment, we have now performed molecular weight analysis and included the molecular weight of our microbial CS in the results section.

Reviewer 3 Comment 3:

3. A more rigorous product characterization process (e.g. purity, NMR results) is needed.

Response to Comment 3: The titers we originally obtained were too low for NMR characterization and we had not attempted to purify the sample. However, in response to this comment, we tried scaling up our cultures and purify the CS. We performed NMR analysis on this small amount of sample (~100 □g) and while we found it did contain CS it also contains other impurities. This process will require substantial scale-up to prepare sufficient amounts of high purity CS required for NMR analysis. We believe we are not yet at the stage where we produce relatively higher product for NMR analysis.

Reviewer 3 Comment 4:

4. Detailed comparison of expression levels and activities of different enzymes under various conditions are lacking.

*Response to Comment 4**:** In response to this comment, we performed SDS-PAGE analysis (presented below) of the lysates expressing different sulfotransferase variants. However, the results were inconclusive and hence we have not included these data in the manuscript. Our original manuscript also contains enzyme activities of the different variants calculated by using the sulfotransferase assay (coupled assay with aryl-sulfotransferase).*

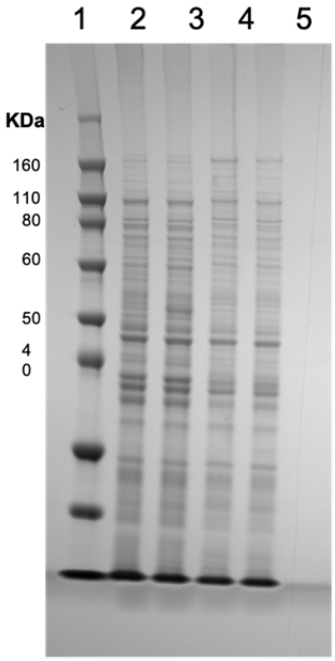

	Key
Lane 1	*Ladder*
Lane 2	*MG1655cysH(DE3) pETM6-PCAFS*$_{W}$
Lane 3	*MG1655cysH(DE3) pETM6-PCAFS*$_{M2}$
Lane 4	k4kfoEcysH(DE3) pETM6-S$_{W}$ pdcas9-dkpsM1
Lane 5	k4kfoEcysH(DE3) pETM6-S$_{M2}$ pdcas9-dkpsM1

Sulfotransferase size: ~ 50 kDa

Reviewer 3 Comment 5:
Some more specific suggestions are shown below:
5. As only CS-A is produced in the report, it is not necessary to include details for CS-B, CS-C, CS-D and CS-E in Fig. 1. In addition, IdoA-containing structure CS-B belongs to dreamt sulfate, not chondroitin sulfate any more.

Response to Comment 5: In response to this comment, we have removed the structures of the other CS types from the diagram.

Reviewer 3 Comment 6:
6. Specific mutations in SM1, SM2, and SM4 should be listed in the main manuscript.

Response to Comment 6: In response to this comment, we have listed the amino acid mutations in each sulfotransferase variant in the main manuscript.

Reviewer 3 Comment 7:
7. More detailed biochemical characterization (expression level, kinetics, etc.) of SW and mutants (SM1, SM2, and SM4) and related results should be shown in the main manuscript or in the Supporting Information file.

Response to Comment 7: In response to the comment, we have performed an SDS PAGE of the lysate. However, performing kinetics for the enzymes is not scalable at its current state of this study and is well beyond the scope of this study. This is a currently ongoing work in our research group.

Reviewer 3 Comment 8:
8. Table S1 should be expanded and moved to the main manuscript. It should include results from the construct with 58% level of CS sulfation, and from different constructs under different conditions investigated in this study.

Response to Comment 8: In response to this comment, we have now included extracellular CS data from our MG1655 CS producing strain to Table S1.

Reviewer #1 (Remarks to the Author):

This revised manuscript has been considerably improved by the additions and corrections made in response to reviewers' comments.

Reviewer #2 (Remarks to the Author):

The current submission is a revised version of a previous manuscript. The extensive revisions greatly improve the manuscript with respect to clarity and brevity of the data presented. However, some mostly stylistic/grammar/spelling issues remain.

-Line 19: Animal issues ?
-Sentence starting on line 19 is awkward
-Line 29 "Overall, this is the first time the simple, one-step microbial production of a sulfated GAG has been demonstrated and marks an important milestone in the animal-free production of these molecules." Suggestion: This is the first demonstration of a one-step………
-Line 57: inhomogeneity OR heterogenous?
-Line 69: "…..have been shown to…." To "…was shown…."
-Line 76: "Chemical synthesis methods are not only tedious and involve multiple steps, but are also difficult to scale up." Suggestion: Chemical synthesis methods are tedious, involving multiple steps and difficult to scale up."
-Line 77 to 79: Awkward sentence
-Line 86: Awkward sentence: Pathway precursors/intermediates are not "tuned". Their level of synthesis are.
Fig 1. Maybe show PAPS. Also, text refers to 1B before 1A. Flip?
-Line 102: This sentence might be true for lab studies but not too many examples in "real life". Insulin, lipids (LS9/REG/Genomatica), others?
-Line 111: Transferases of animal origin, as opposed to an animal transferase.
-Line 123: "We also integrated the T7 RNA polymerase into the genomic lacZ site in E. coli K4 ΔkfoE, generating K4 ΔkfoE (DE3), to facilitate expression of target genes under the stronger T7 promoter system originally found in the T7 bateriophage (Supplementary Fig, S1)." Suggestion: "We also integrated the T7 RNA polymerase into the genomic lacZ site in E. coli K4 ΔkfoE, generating K4 ΔkfoE (DE3), to facilitate expression of recombinant genes under the stronger T7 promoter system (Supplementary Fig, S1)."
-Fig 2B: Natural? Maybe wildtype?
-Fig 2: As in Fig 1, Fig 2B is referred before Fig 2A in the text. Flip?
-Line 149: "…..repression and deletion…." Suggestion "…..repression or deletion……"
-Line 151: Awkward sentence
-Line 153: "Moreover, this study points out that due to very low native accumulation, PAPS pathway intervention is a necessary condition for GAG biosynthesis in E. coli." Suggestion: "This result shows that PAPS pathway intervention is a necessary condition for GAG biosynthesis in E. coli."
-Line 155-156: Eliminate the sentence. It overstated throughout the manuscript.
Line 157: Be precise. As a result of kfoE and cysH deletions and Sw over-expression, CS sulfation……
Line 158: Question: Was over-expression of cysDN alone attempted? It commits ATP to PAPS synthesis no?
-Line 169: Fig 2D?
-Line 182: "……PROSS that is based on the previously elucidated structure of a similar protein. Specifically, the sulfotransferase domain of an olefin synthase (from Synechococcus PCC 7002) was used as the template to model Sw (RMSD of 0.74)." Suggestion: "……PROSS that is based on the previously elucidated structure of the sulfotransferase domain of an olefin synthase from Synechococcus PCC 7002 (RMSD of 0.74)."
-Fig 3B and Fig 3C represent the same data no? Remove Fig 3C.
-Line 206: "The resulting…." Remove this sentence.

-Line 207: “Interestingly, …….” Get rid of all of these (there is another on line 264). They are very subjective. What is interesting to you may not be to those and they just take up space.
-Line 224: awkward sentence starting with “To explore…..
-Line 236: were fermentation conditions improved or protein expression? Or one led to the other?
Line 259: “Five spacers targeting kpsT and kpsM on the genome of K4 strain were chosen to repress GAG transport (Supplementary Fig. S4). These were designated as dT1, dT2, dT3, dM1 and dM2. They were cloned into the CRISPRi plasmid pdCas9, transformed into E. coli K4ΔkfoEΔcysH(DE3) pETM6-Sw.” Suggestion: “Five spacers targeting kpsT and kpsM, designated as dT1, dT2, dT3, dM1 and dM2, were chosen to repress GAG transport (Supp Fig 4A). Based on XXXXXX (Supp Fig 4B), the strain obtained by transformation with pdCas9-dM1 was chosen for further investigating the……..”
-Line 259: Question: Are these gene in an operon? If so, is M1 lowering expression of both genes?
-Line 276: This section opens up a can of worms that should probably be left closed for this study. That is how import is a lot of the result reported here once the strain is moved into a bioreactor. It is often the case that shake flask results do not translate to bioreactors. I would seriously consider removing this data and saving it for another paper.

The discussion is an improvement from the first submission but in my opinion falls short of a critical comparison to previously published studies.

First 2 sentences need citations.
-Line 353: is 55% really comparable to 70%? If so why?

Reviewer #3 (Remarks to the Author):

The revised manuscript has successfully addressed some of the concerns that I had previously. I support the acceptance of its publication.

Response to Reviewers' Comments

Reviewer #1 (Remarks to the Author):

This revised manuscript has been considerably improved by the additions and corrections made in response to reviewers' comments.

Reviewer #2 (Remarks to the Author):

The current submission is a revised version of a previous manuscript. The extensive revisions greatly improve the manuscript with respect to clarity and brevity of the data presented. However, some mostly stylistic/grammar/spelling issues remain.

Reviewer 2 Comments 1:
-Line 19: Animal issues ?
-Sentence starting on line 19 is awkward
-Line 29 "Overall, this is the first time the simple, one-step microbial production of a sulfated GAG has been demonstrated and marks an important milestone in the animal-free production of these molecules." Suggestion: This is the first demonstration of a one-step………
-Line 57: inhomogeneity OR heterogenous?
-Line 69: "…..have been shown to…." To "…was shown…."
-Line 76: "Chemical synthesis methods are not only tedious and involve multiple steps, but are also difficult to scale up." Suggestion: Chemical synthesis methods are tedious, involving multiple steps and difficult to scale up."
-Line 77 to 79: Awkward sentence
-Line 86: Awkward sentence: Pathway precursors/intermediates are not "tuned". Their level of synthesis are.
-Fig 1. Maybe show PAPS. Also, text refers to 1B before 1A. Flip?
-Line 102: This sentence might be true for lab studies but not too many examples in "real life". Insulin, lipids (LS9/REG/Genomatica), others?
-Line 111: Transferases of animal origin, as opposed to an animal transferase.
-Line 123: "We also integrated the T7 RNA polymerase into the genomic lacZ site in E. coli K4 ΔkfoE, generating K4 ΔkfoE (DE3), to facilitate expression of target genes under the stronger T7 promoter system originally found in the T7 bateriophage (Supplementary Fig, S1)." Suggestion: "We also integrated the T7 RNA polymerase into the genomic lacZ site in E. coli K4 ΔkfoE, generating K4 ΔkfoE (DE3), to facilitate expression of recombinant genes under the stronger T7 promoter system (Supplementary Fig, S1)."
-Fig 2B: Natural? Maybe wildtype?
-Fig 2: As in Fig 1, Fig 2B is referred before Fig 2A in the text. Flip?
-Line 149: "…..repression and deletion…." Suggestion "…..repression or deletion……"
-Line 151: Awkward sentence
-Line 153: "Moreover, this study points out that due to very low native accumulation, PAPS pathway intervention is a necessary condition for GAG biosynthesis in E. coli." Suggestion:

“This result shows that PAPS pathway intervention is a necessary condition for GAG biosynthesis in E. coli.”
-Line 155-156: Eliminate the sentence. It overstated throughout the manuscript.
Line 157: Be precise. As a result of kfoE and cysH deletions and Sw over-expression, CS sulfation……

Response to Reviewer's comments: *We thank the reviewer for these carefully observed suggestions. All the sentences mentioned above have been modified accordingly.*

Reviewer 2 Comment 2:
Line 158: Question: Was over-expression of cysDN alone attempted? It commits ATP to PAPS synthesis no?

Response to Reviewer's comments*: Yes, we attempted overexpression of different combinations of the PAPS biosynthetic pathway and observed that only overexpression of cysDNC and cysDNCQ resulted in an observable increase in PAPS synthesis. This is published as part of our previous manuscript (Badri et al. (2019) Biotechnology J)*

Reviewer 2 Comments 3:
-Line 169: Fig 2D?
-Line 182: “……PROSS that is based on the previously elucidated structure of a similar protein. Specifically, the sulfotransferase domain of an olefin synthase (from Synechococcus PCC 7002) was used as the template to model Sw (RMSD of 0.74).”
Suggestion: “……PROSS that is based on the previously elucidated structure of the sulfotransferase domain of an olefin synthase from Synechococcus PCC 7002 (RMSD of 0.74).”
-Fig 3B and Fig 3C represent the same data no? Remove Fig 3C.
-Line 206: “The resulting….” Remove this sentence.
-Line 207: “Interestingly, …….” Get rid of all of these (there is another on line 264). They are very subjective. What is interesting to you may not be to those and they just take up space.
-Line 224: awkward sentence starting with “To explore…..
Line 259: “Five spacers targeting kpsT and kpsM on the genome of K4 strain were chosen to repress GAG transport (Supplementary Fig. S4). These were designated as dT1, dT2, dT3, dM1 and dM2. They were cloned into the CRISPRi plasmid pdCas9, transformed into E. coli K4ΔkfoEΔcysH(DE3) pETM6-Sw.” Suggestion: “Five spacers targeting kpsT and kpsM, designated as dT1, dT2, dT3, dM1 and dM2, were chosen to repress GAG transport (Supp Fig 4A). Based on XXXXXX (Supp Fig 4B), the strain obtained by transformation with pdCas9-dM1 was chosen for further investigating the……..”

Response to Reviewer's comments: *We thank the reviewer for these carefully observed suggestions. All the sentences mentioned above have been modified accordingly.*

Reviewer 2 Comment 4:

-Line 236: were fermentation conditions improved or protein expression? Or one led to the other?

***Response to Reviewer's comments**: Thank you for this observation. In response, we now have changed the sentence to say "induction" conditions were optimized.*

Reviewer 2 Comment 5:

-Line 259: Question: Are these gene in an operon? If so, is M1 lowering expression of both genes?

***Response to Reviewer's comments**: Yes, the genes kpsM and kpsT appear to be in an operon format. While we did not probe into the details, we believe employing spacer dM1 could have repressed both kpsM and kpsT.*

Reviewer 2 Comment 6:

-Line 276: This section opens up a can of worms that should probably be left closed for this study. That is how import is a lot of the result reported here once the strain is moved into a bioreactor. It is often the case that shake flask results do not translate to bioreactors. I would seriously consider removing this data and saving it for another paper.

***Response to Reviewer's comments**: Thank you for this comment. We agree that the shake flask results do not translate to fermentations and this was observed in our experiment. For these same reasons, we feel it is important to keep this section and demonstrate this observation.*

Reviewer 2 Comment 7:

The discussion is an improvement from the first submission but in my opinion falls short of a critical comparison to previously published studies. First 2 sentences need citations.

***Response to Reviewer's comments**: In response to this comment, we have not added citations for the first two sentences. Additionally, we have increased the relevant citations in the discussion section from 7 to 16.*

Reviewer 2 Comment 8:

-Line 353: is 55% really comparable to 70%? If so why?

***Response to Reviewer's comments**: In response to this comment, we have now replaced the word "comparable" with "similar".*

Reviewer #3 (Remarks to the Author):

The revised manuscript has successfully addressed some of the concerns that I had previously. I support the acceptance of its publication.
